# Functional profiling of somatostatin receptors identifies somatostatin receptor subtype 2 as a vulnerability in Succinate Dehydrogenase *SDHB*-deficient pheochromocytomas and paragangliomas

**DOI:** 10.1186/s43556-026-00440-5

**Published:** 2026-04-03

**Authors:** Víctor García-Vioque, Sergio Pedraza-Arevalo, María Trinidad Moreno-Montilla, Esther Rivero-Cortés, Ricardo Blázquez-Encinas, Federica Mangili, Ester Arroba, Aura D. Herrera-Martínez, Michael D. Culler, María Ángeles Gálvez-Moreno, Anne Barlier, Luisa María Botella, Mercedes Robledo, Justo P. Castaño, Alejandro Ibáñez-Costa

**Affiliations:** 1 Department of Cell Biology, Physiology, and Immunology, University of Cordoba, Cordoba, Spain; 2 Maimonides Biomedical Research Institute of Cordoba (IMIBIC), Cordoba, Spain; 3 Reina Sofia University Hospital (HURS), Cordoba, Spain; 4 SC Endocrinology, Fondazione IRCCS Ca’ Granda Ospedale Maggiore Policlinico Di Milano, Milan, Italy; 5 Hereditary Endocrine Cancer Group, Human Cancer Genetics Program, Spanish National Cancer Research Centre (CNIO), Madrid, Spain; 6 Department of Endocrinology and Nutrition, Reina Sofia University Hospital, Cordoba, Spain; 7 IPSEN Bioscience, Cambridge, MA USA; 8 Aix Marseille Univ, APHM, INSERM, MMG U1251, La Timone University Hospital, Laboratory of Molecular Biology GEnOPé, BIOGENOPOLE, Marseille, France; 9 Centro de Investigaciones Biológicas, Margarita Salas | CSIC, Madrid, Spain; 10 Centro de Investigación Biomédica en Red de Enfermedades Raras, Madrid, Spain; 11 CIBER Physiopathology of Obesity and Nutrition (CIBERobn), Cordoba, Spain

**Keywords:** Pheochromocytoma, Paraganglioma, Somatostatin, Somatostatin Receptor 2, *SDHB*

## Abstract

Pheochromocytomas and Paragangliomas (PPGL) are rare neuroendocrine tumors with favorable prognosis, although a significant subset (20–25%) progress to metastasis, worsening patient prognosis. For metastatic cases, pharmacological interventions become essential, yet most tumors show poor response to treatment. While clinical trials are ongoing, there is no established treatment for metastatic PPGL. Like other neuroendocrine tumors, PPGL exhibit high membrane expression of somatostatin receptors, and despite Peptide Receptor Radionuclide Therapy, PRRT, strategies have successfully been implemented, trials with cold somatostatin analogs were abandoned prematurely due to inconsistent results. To investigate this issue and identify potential therapeutic tools, we widely profiled somatostatin receptors expression in PPGL and conducted a comprehensive functional screening on wild-type and *SDHB* knockdown PPGL cell lines of native and synthetic somatostatin analogs. Results revealed that pheochromocytomas and paragangliomas similarly display a predominant *SSTR2* and *SSTR1* expression regardless of molecular cluster. Treatment with somatostatin, cortistatin, octreotide or pasireotide did not exert clear antitumoral effects on model cell lines. Notably, the selective SST_2_ agonist BIM-23120 significantly reduced cell proliferation and induced apoptosis in an SST_2_-dependent manner, but only in *SDHB* knocked-down PPGL cells. Indeed, only *SDHB* KD cells showed stronger membrane-enriched SST_2_ and clear receptor internalization upon BIM-23120 treatment. Molecular analysis revealed a generalized dephosphorylation affecting key proliferation, growth and cell survival pathways in response to BIM-23120 (unlike when treating with octreotide). Altogether, our results provide novel information on the status of the somatostatin system in PPGL and identify new potential therapeutic tools selectively targeting somatostatin receptors on this refractory tumor.

**Supplementary Information:**

The online version contains supplementary material available at 10.1186/s43556-026-00440-5.

## Introduction

Pheochromocytomas and Paragangliomas (PPGL) are rare Neuroendocrine Neoplasms (NENs) derived from chromaffin cells of the adrenal medulla and neural crest progenitors of the extra-adrenal paraganglia, respectively [1]. Although classically considered benign, their significant probability of metastasis and recurrence prompted the WHO to reclassify them as malignant neoplasms with variable metastatic potential [2]. Accordingly, all patients with PPGL are considered to have a lifelong risk of metastases [3]. Multi-omics studies have established the existence of three distinct molecular clusters with different prognosis: pseudohypoxic, kinase signaling-altered and Wnt-altered groups [4]. Germline and somatic mutations in more than 20 susceptibility genes occur in ~ 70% of all patients with PPGL [5], with tumors harboring Succinate Dehydrogenase Complex Iron Sulfur Subunit B (*SDHB*) germline mutations, classified within the pseudohypoxia-related cluster, bearing the highest metastatic risk [6].

Surgical resection stands as the only curative option for PPGL [1]. When surgery is not feasible, alternative treatments such as radiotherapy, chemotherapy, tyrosine kinase inhibitors or radionuclide therapy are applied [7]. Unfortunately, despite substantial advances in understanding the molecular landscape of PPGL and metastatic PPGL (mPPGL) over the past decade [4, 8, 9], effective translation into targeted therapeutic strategies remains limited.

A hallmark of most NENs, particularly Gastroenteropancreatic NENs (GEP-NENs), is the high expression of Somatostatin Receptors (SSTs), which lends them responsive to treatment with synthetic Somatostatin Analogs (SSAs) [10–12]. Somatostatin and its relative peptide, cortistatin, bind with high affinity to their five SSTs (SST_1_-SST_5_), encoded by five separate genes (*SSTR1*-*SSTR5*) [13]. Octreotide and lanreotide, the first SSAs successfully developed as clinical tools, preferentially target SST_2_, and have demonstrated their antisecretory and antiproliferative effect in NENs [11, 12]. Subsequent search for more universal analogs led to the development of the so-called second-generation analog, pasireotide, which binds SST_5_ but also SST_2_ and SST_1_ with high affinity [14].

Consistent with findings in GEP-NENs, early studies reported SSTs overexpression in PPGL [15], with predominant *SSTR2* expression and lower levels of *SSTR1,* and very low levels of *SSTR3* and *SSTR5*, highlighting a molecular similarity between Pheochromocytomas (PCC) and Paragangliomas (PGL) [16]. Intriguingly, unlike in other NENs, the first efficacy tests of SSAs on PPGL were unsuccessful, owing to the absence of conclusive results in terms of therapeutic benefits, which led to premature abandonment of the application of SSAs in PPGL [17]. This may explain the paucity of clinical trials, with only one trial, currently underway, prospectively evaluating lanreotide in mPPGL (LAMPARA, NCT03946527), and hence, of data related on the impact of cold SSAs on PPGL [18]. In contrast, the use of radiolabeled analogs has enabled the successful application of Peptide Receptor Radionuclide Therapy (PRRT) to treat PPGL [19].

The mechanisms underlying the limited efficacy of cold SSAs in PPGL, despite strong SSTs expression, remain unclear. Existing studies, mainly focused on octreotide’s effects on catecholamine secretion and blood pressure, report inconsistent findings and lack in-depth analysis of antitumoral properties [20, 21]. These studies are further limited by small sample sizes [22]. To address this, we characterized SSTs expression in two PPGL tumor cohorts and validated findings using public datasets. Functional assays using two established PPGL cell models evaluated the effects of somatostatin, cortistatin, clinically used SSAs, and experimental SSTs subtype-selective agonists. Our findings offer novel insights into SST signaling and highlight distinct activity of an SST_2_-selective agonist, particularly in *SDHB*-deficient cells.

## Results

### SSTs expression pattern was consistent and similar in human PPGL samples

**Fig. 1:**
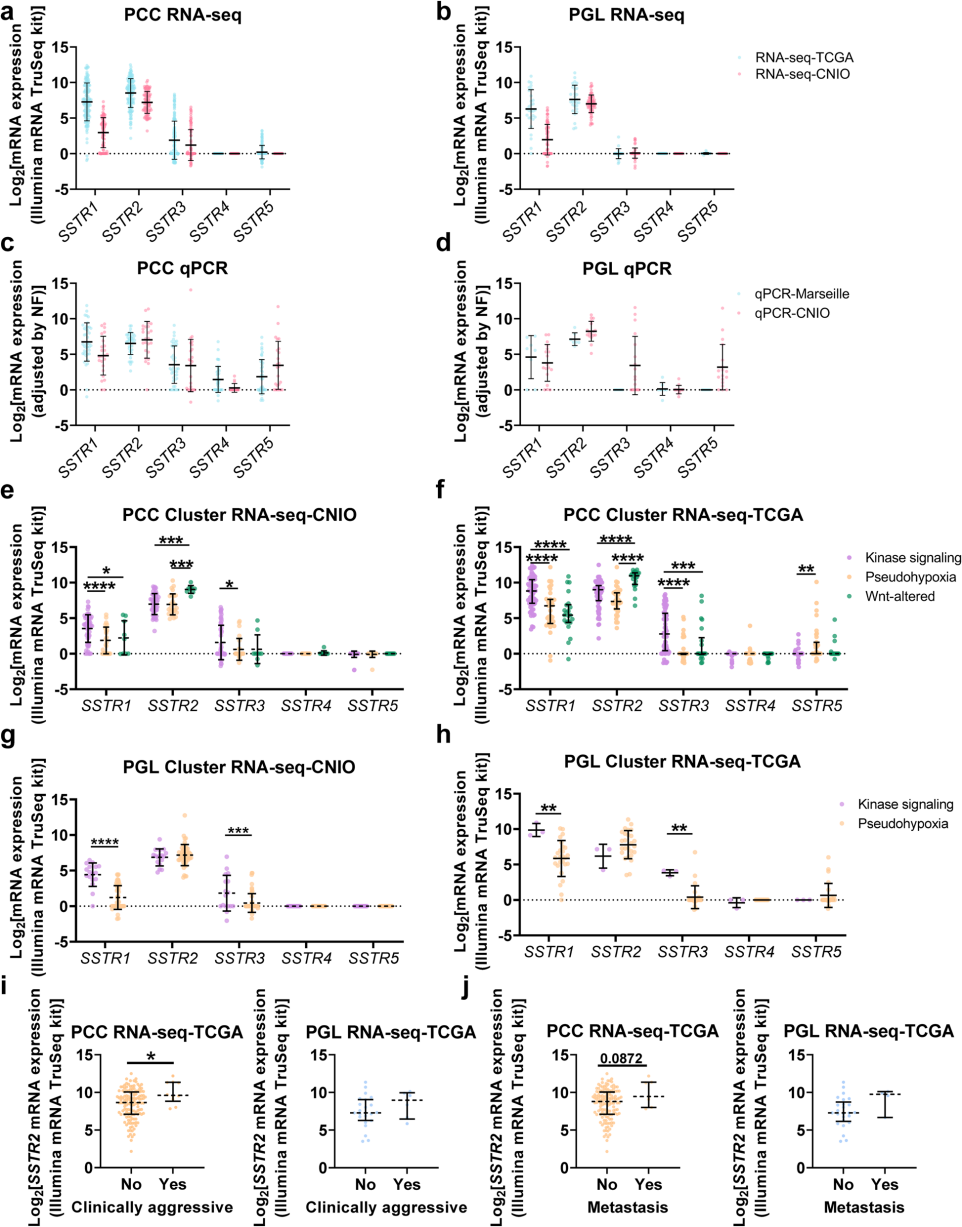
Expression profile of SSTs at RNA level and association with clinical parameters in PPGL. **a**-**d** Relative mRNA expression of SSTs in PCC and PGL in the RNA-seq cohorts and in the qPCR cohorts. **e**–**h** Relative mRNA expression of SSTs depending on the molecular cluster of neoplasms in PCC and PGL in the CNIO and the TCGA RNA‑seq cohorts. Receptor expression is shown in purple for those samples belonging to the kinase signaling cluster, in orange for those belonging to the pseudohypoxia cluster, and in green for those belonging to the Wnt-altered group. **i**, **j** Association of relative *SSTR2* mRNA expression with clinically aggressive behavior and metastasis in PCC (orange) and PGL (blue) in the TCGA cohort. Median and interquartile range are represented. Asterisks indicate significant differences between groups (* *p* < 0.05, ** *p* < 0.01, *** *p* < 0.001, and **** *p* < 0.0001)

To characterize the expression landscape of SSTR subtypes in PCC and PGL, mRNA levels were analyzed across independent qPCR (qPCR-CNIO, qPCR-Marseille) and RNA-seq (RNA-seq-CNIO, RNA-seq-TCGA) cohorts. *SSTR2* and *SSTR1* were consistently highly expressed in PCC and PGL, whereas *SSTR3* showed lower levels, and *SSTR4* and *SSTR5* levels were minimal or undetectable. The qPCR data confirmed RNA-seq trends for *SSTR1* and *SSTR2*, whereas some conspicuous variations were observed for the less expressed subtypes, particularly *SSTR5* and *SSTR4* in PCC across both cohorts and in PGL for the qPCR-CNIO cohort (Fig. 1a-d). Receptor expression varied across molecular clusters in both cohorts. In PCC, *SSTR1* and *SSTR3* were elevated in the kinase signaling cluster, *SSTR2* in the Wnt pathway cluster, and *SSTR5* in pseudohypoxic cluster in the RNA-seq-TCGA cohort (Fig. 1e and f). PGL showed a similar pattern, where *SSTR1* and *SSTR3* showed higher expression in kinase signaling cluster samples (Fig. 1g and h).

We next focused on potential relationships between receptor expression levels and key clinical parameters. In the RNA-seq-TCGA cohort, higher *SSTR2* expression was observed in clinically aggressive tumors, defined by the occurrence of distant metastases, positive regional lymph nodes, or local recurrence, and metastases samples, although the association remained significant only for clinically aggressive PCC (Fig. 1i and j) perhaps due to the low number of metastatic PCC. In contrast, no clear associations were found for the other receptors analyzed (Fig. S1a-d), except for *SSTR1* in PGL, where higher expression was observed in samples from patients without metastasis (Fig. S1b).

To examine SSTRs expression in comparison with normal tissue, we analyzed 295 normal adrenal gland tissue samples from GTEx, although adrenal tissue is not an ideal control for PPGL. In this comparison, *SSTR1* and *SSTR2* were significantly overexpressed in both PCC and PGL relative to normal tissue. *SSTR3* was upregulated in PCC but downregulated in PGL. Conversely, *SSTR4* showed significantly higher expression in normal tissue compared with PCC, and *SSTR5* exhibited higher expression in normal tissue than in PCC and PGL (Fig. S1e).

To further examine potential molecular associations in these tumor types, we analyzed the expression of 48 genes downstream of SST_2_ activation (Supp. Table 1) using transcriptomic data from CNIO (Fig. S2a and b) and TCGA RNA-seq cohorts (Fig. S2c and d). Expression levels of SST_2_-associated genes effectively distinguished between the kinase signaling and pseudohypoxia clusters (Fig. S2), whereas the few samples classified in the Wnt signaling cluster were scattered between them. PLS-DA analyses identified 15 key genes driving this separation, with six— *NOS2*, *ADCY1*, *MAPK3*, *PLAGL1*, *CDK2*, and *HRAS*— shared across both cohorts (Fig. S2b and d).

Overall, these data demonstrate that *SSTR1* and *SSTR2* are consistently overexpressed in PCC and PGL and associate with distinct molecular and clinical features, underscoring their potential relevance as biomarkers and therapeutic targets in PPGL.

### Natural peptides, clinically available SSAs, and specific SSTs agonists exert distinct effects on functional parameters in PPGL cell lines

**Fig. 2:**
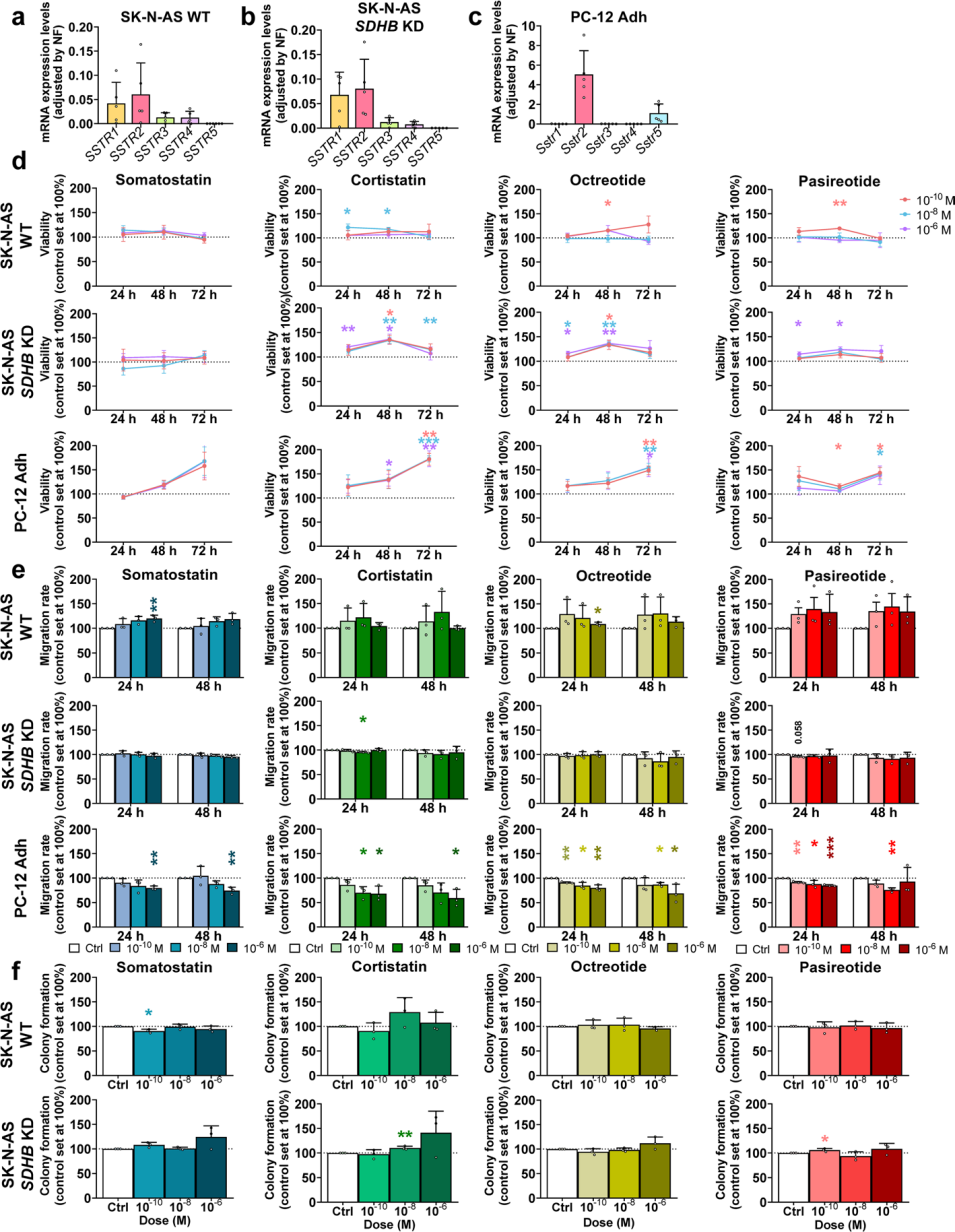
In vitro response of PPGL cell models to treatment with classical SSAs. **a**-**c** Relative mRNA expression levels of SSTRs in the cell lines SK-N-AS WT, SK-N-AS *SDHB* KD and PC-12 Adh (*n* = 5). **d** Changes in cell viability at 24, 48 and 72 h in response to classical SSAs in the human cell lines SK-N-AS WT and *SDHB*-altered and the rat line PC-12 Adh (*n* = 3). **e** Migration rate of the three cell lines employed treated with classical SSAs (*n* = 3). **f** Colony-forming capacity of human lines in response to the mentioned molecules (*n* = 3). Data represent mean ± SD. Colored asterisks indicate statistically significant differences compared with the control group. Black asterisks above brackets denote statistically significant differences between the indicated groups (* *p* < 0.05, ** *p* < 0.01, *** *p* < 0.001)

To examine the functional actions of somatostatin and its analogs in PPGL, three representative cell lines were used, WT SK-N-AS, *SDHB*-silenced SK-N-AS and PC-12 Adh. All cell lines displayed a SSTs mRNA pattern consistent with human tumors, with high *SSTR2* and *SSTR1* expression and low or negligible levels of the rest of the receptors, particularly *SSTR5* in SK-N-AS cells, which showed a nearly identical profile (Fig. 2a-c).

Treatment with somatostatin, cortistatin, octreotide or pasireotide at various doses (10^–6^ to 10^–10^ M) and increasing times (24–72 h) did not result in any significant reduction on cell viability. Instead, significant stimulatory effects were observed for cortistatin in the three cell lines, and, after long incubation times, in PC-12 Adh cells (Fig. 2d).

We next assessed the actions of these molecules on cell migration and colony formation. All tested compounds significantly reduced PC-12 Adh migration, with octreotide and pasireotide showing the strongest effects, even at lower doses and shorter incubation times (Fig. 2e). Somatostatin and cortistatin also exerted inhibitory impacts on migration of these cells, although at higher concentration. In contrast, human cell lines showed minimal responses to treatment, with only minor, inconsistent effects, such as slight inhibition by 10^–8^ M cortistatin at 24 h in SK-N-AS *SDHB* Knocked-Down (KD) cells and a stimulatory effect for high-dose somatostatin and octreotide at 24 h in SK-N-AS WT cells. Colony formation was largely unaffected, with only subtle, opposing changes observed in SK-N-AS variants (Fig. 2f). This parameter could not be quantified in PC-12 Adh cells owing to its weak crystal violet staining.

The overall lack of solid responses prompted us to test alternative specific agonists with selective affinity for each receptor subtype, except for SST_4_, which is practically absent in all NENs. Importantly, unlike previously tested compounds, these agonists exhibit a higher affinity for a single receptor, with differences of several orders of magnitude compared to the next receptor for which they have high affinity, as shown in Supp. Table 2. Specifically, we employed the agonists BIM-23926, BIM-23120, BIM-355 and BIM-23206, which are selective for SST_1_, SST_2_, SST_3_ and SST_5_, respectively [13].

**Fig. 3:**
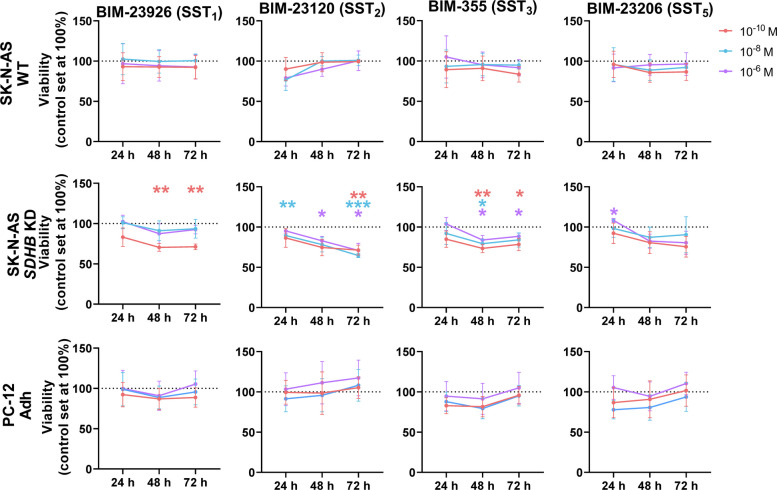
Effect of treatment with selective agonists on cell viability in PPGL cell lines. Changes in cell viability at 24, 48 and 72 h in response to the selective agonists in the human cell lines SK-N-AS WT, *SDHB*-altered and the rat line PC-12 Adh (*n* = 3). Data represent mean ± SD. Colored asterisks indicate statistically significant differences compared with the control group (* *p* < 0.05, ** *p* < 0.01, *** *p* < 0.001)

Similarly to the classical ligands, the selective agonists did not induce any change in cell viability for SK-N-AS WT and PC-12 Adh cells (Fig. 3). However, in the *SDHB*-silenced SK-N-AS, compounds selective for SST_1_, SST_3_, and, particularly, SST_2_ evoked substantial reductions of cell viability. While the effects did not seem to follow a clear dose-related pattern, they were clearly observable and showed a time-dependent profile, being most prominent at 48 and 72 h. Specifically, BIM-23926 (10^–10^ M) reduced viability by 30% at 48 h, BIM-23120 (10^–8^ M) by 35% at 72 h, and BIM-355 (10^–8^ M) by 26% at 48 h. Notably, whereas the viability reductions caused by BIM-23926 and BIM-355 at 48 h were mostly sustained at 72 h, BIM-23120 progressively decreased viability throughout the entire treatment period.

**Fig. 4:**
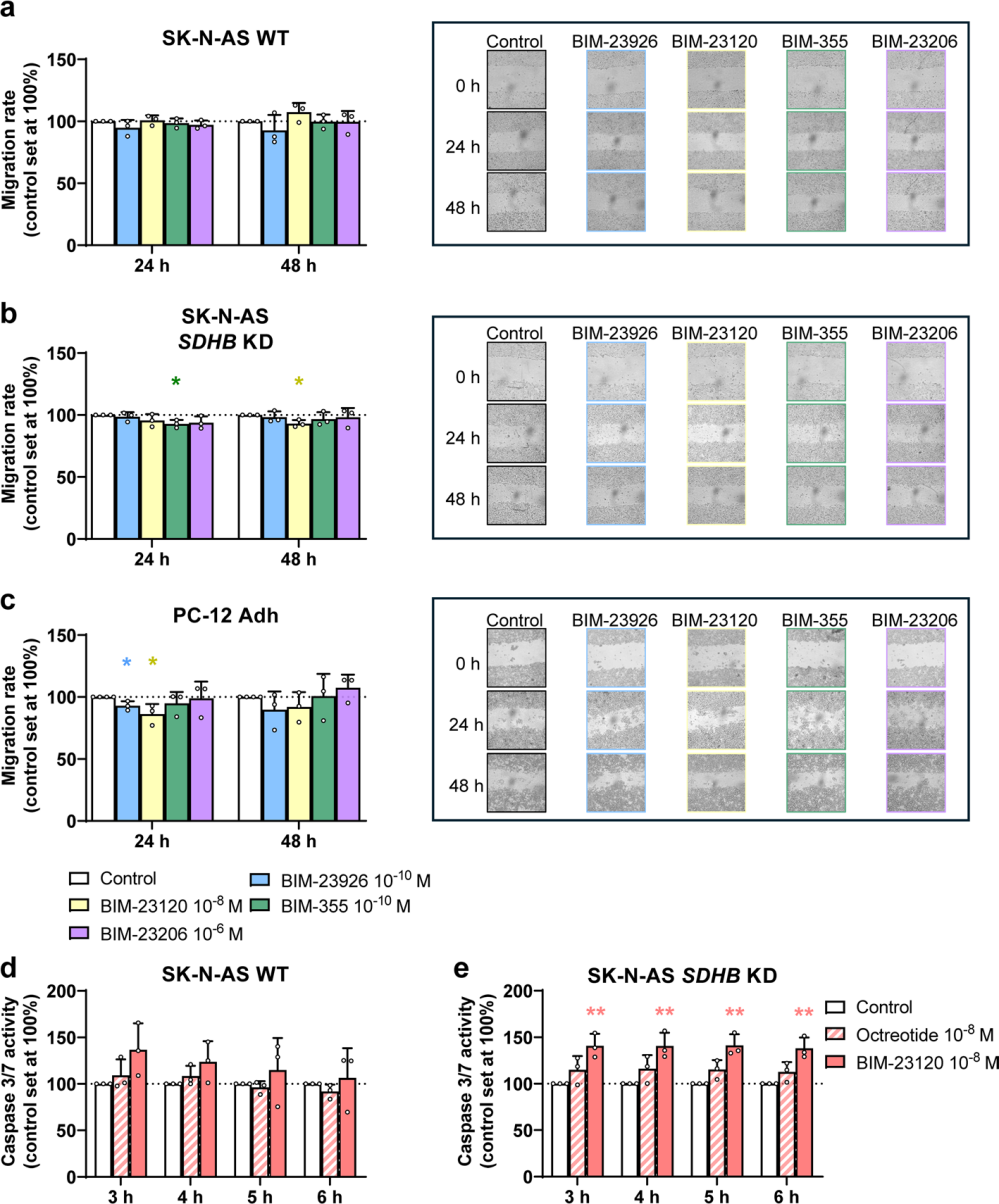
Effect of treatment with selective analogs on migration and apoptosis in PPGL cell lines. **a**-**c** Migration rate of the SK-N-AS WT, SK-N-AS *SDHB* KD and PC-12 Adh cells in response to the drugs mentioned above (*n* = 3); representative images of the lines that showed statistically significant changes are included. **d**, **e** Apoptosis assay in the SK-N-AS WT and SK-N-AS *SDHB* KD cell lines in response to treatment with octreotide (red and white) and BIM-23120 (red) at 3, 4, 5 and 6 h (*n* = 3). Data represent mean ± SD. Colored asterisks indicate statistically significant differences compared with the control group (* *p* < 0.05, ** *p* < 0.01)

On the other hand, the effects evoked on migration and clonogenicity, were, overall, much more limited (Fig. 4a-c, Fig. S3a and b). Specifically, for each compound we selected doses with the maximal effect on cell proliferation, but significant reductions in cell migration were observed only for BIM-23926, BIM-23120 and BIM-355 in the SK-N-AS *SDHB* KD and PC-12 Adh lines, though none exceeded a 12% decrease (Fig. 4a-c). Similarly, colony formation was significantly reduced only by 10^–6^ M BIM-23206 and with a modest 8% decrease (Fig. S3a and b).

Collectively, these findings indicate that somatostatin and its analogs exert limited functional effects in PPGL-derived cell models. However, selective activation of individual receptor subtypes, particularly SST_2_ in *SDHB*-deficient cells, elicits measurable antiproliferative responses, underscoring receptor- and genotype-dependent signaling differences.

### Cell- and treatment-dependent alterations on cell apoptosis

Subsequent studies were focused on SST_2_ in tumors (Fig. 1a-d) and cell models (Fig. 2a-c), as the main target for drugs approved for NEN treatment. Consistently, its selective agonist BIM-23120 exerted the most significant effects on cell viability in the present study (Fig. 3a). Accordingly, we compared the actions of BIM-23120 and octreotide in apoptosis (both at 10^–8^ M), in WT and *SDHB*-silenced SK-N-AS cells (Fig. 4d and e). In line with cell viability assays, octreotide did not exert any significant effect in apoptosis in any of the two cell models. Conversely, BIM-23120 triggered a significant rise in apoptosis exclusively in *SDHB*-silenced SK-N-AS cells, as compared to the control (> 40% higher caspase activity).

These findings demonstrate that selective SST_2_ activation by BIM-23120 robustly induces apoptosis in *SDHB*-deficient SK-N-AS cells, whereas octreotide remains ineffective, emphasizing the critical role of receptor-specific signaling in mediating apoptotic responses in PPGL models.

### The SST_2_-selective agonist BIM-23120 is not potentiated by combination therapies

Despite the apparent efficacy of BIM-23120 as an antiproliferative agent in *SDHB*-silenced SK-N-AS cells, its impact on clonogenicity or migration was moderate. Therefore, we sought to determine whether its combination with antitumor agents, such as the mTOR inhibitor everolimus or the antiangiogenic drug sunitinib, could potentiate its effects.

**Fig. 5:**
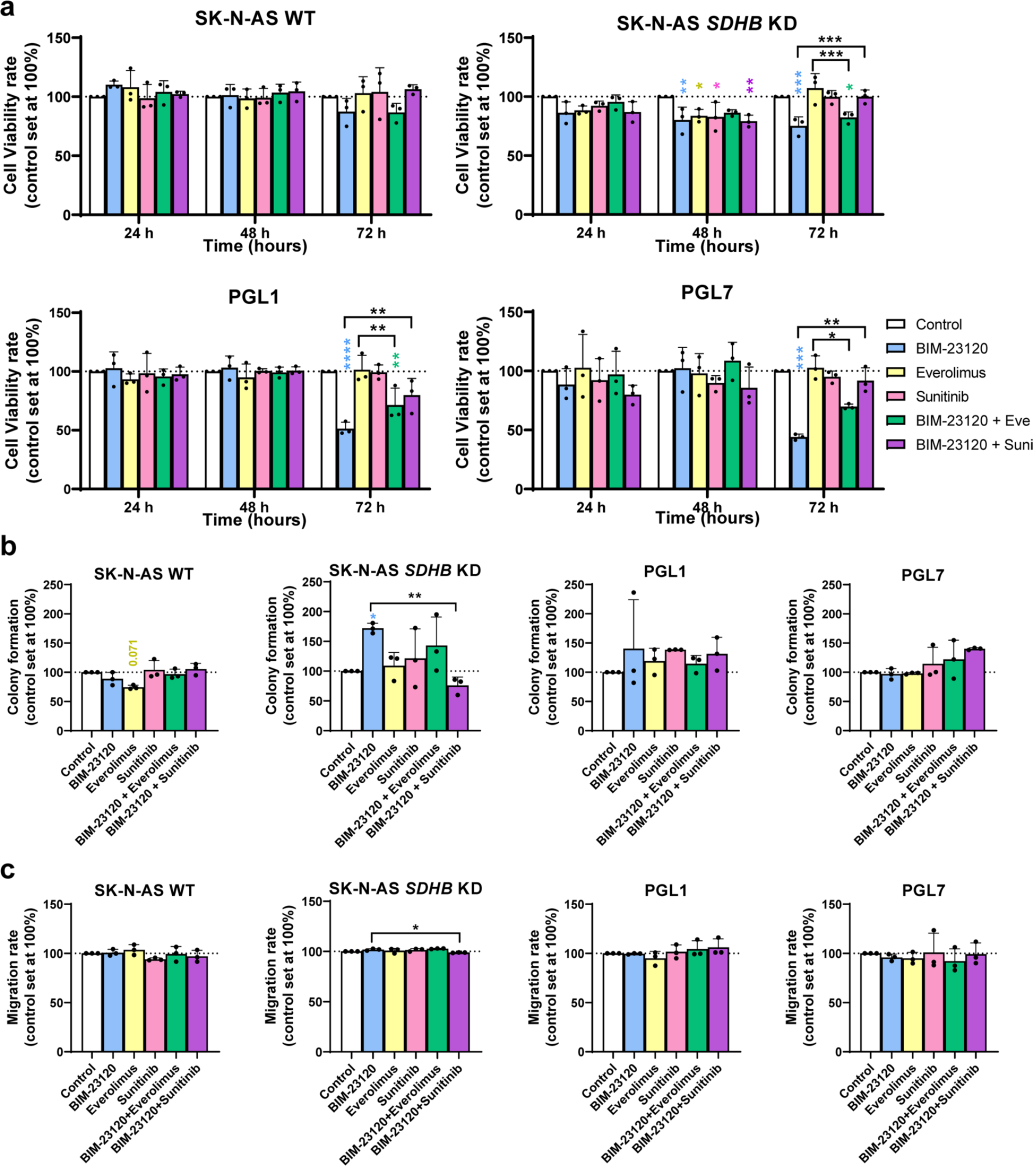
In vitro response of PPGL cell models to treatment with BIM-23120 alone and in combination with other drugs. **a** Changes in cell viability at 24, 48 and 72 h in response to BIM-23120 alone and in combination with other drugs in the human cell lines SK-N-AS WT and *SDHB*-altered and the PGL-derived cells PGL1 and PGL7 (*n* = 3). **b** Colony-forming capacity of human lines in response to the mentioned molecules (*n* = 3). **c** Migration rate of the four cell models employed treated with BIM-23120 alone and in combination with other drugs (*n* = 3). Data represent mean ± SD. Colored asterisks indicate statistically significant differences compared with the control group. Black asterisks above brackets denote statistically significant differences between the indicated groups (* *p* < 0.05, ** *p* < 0.01, *** *p* < 0.001 and **** *p* < 0.0001)

Regarding cell viability, none of the treatments affected WT SK-N-AS cells, either alone or in combination with BIM-23120, consistent with our previous observations, which reinforces the treatment unresponsive profile of this model. In contrast, in *SDHB*-silenced SK-N-AS cells, BIM-23120 significantly reduced viability at 48 and 72 h, while everolimus and sunitinib alone also decreased viability at 48 h, although this effect was lost at 72 h. The effects of treatment with single drugs was not further enhanced by combined treatments at 48 h. In fact, upon longer incubation the inhibitory action of BIM-23120 was still observed whereas that of everolimus or sunitinib monotherapy was not, and in combined treatments only BIM-23120 with everolimus remained significant. In PGL1 and PGL7 cells, only BIM-23120 significantly decreased cell viability at 72 h, whereas combined therapies did not enhance the effect of the agonist alone or even counteracted it (Fig. 5a).

With respect to clonogenic potential, no significant effects were observed in WT SK-N-AS cells. In *SDHB*-silenced SK-N-AS cells, only BIM-23120 treatment led to a paradoxical increase in clonogenicity, which was counteracted by combination with sunitinib. In PGL1 and PGL7 cells, no appreciable effects were detected (Fig. 5b). Likewise, none of the treatments tested, either alone or in combination, significantly altered migration in SK-N-AS or PGL cell lines (Fig. 5c).

Overall, these results indicate that, in the experimental settings and models tested, BIM-23120 exerts the most noticeable antitumor effects, which were not appreciably improved when applied in combination with everolimus or sunitinib.

### BIM-23120 inhibited key pathways associated with cell growth, proliferation, and survival in *SDHB*-silenced cells

**Fig. 6:**
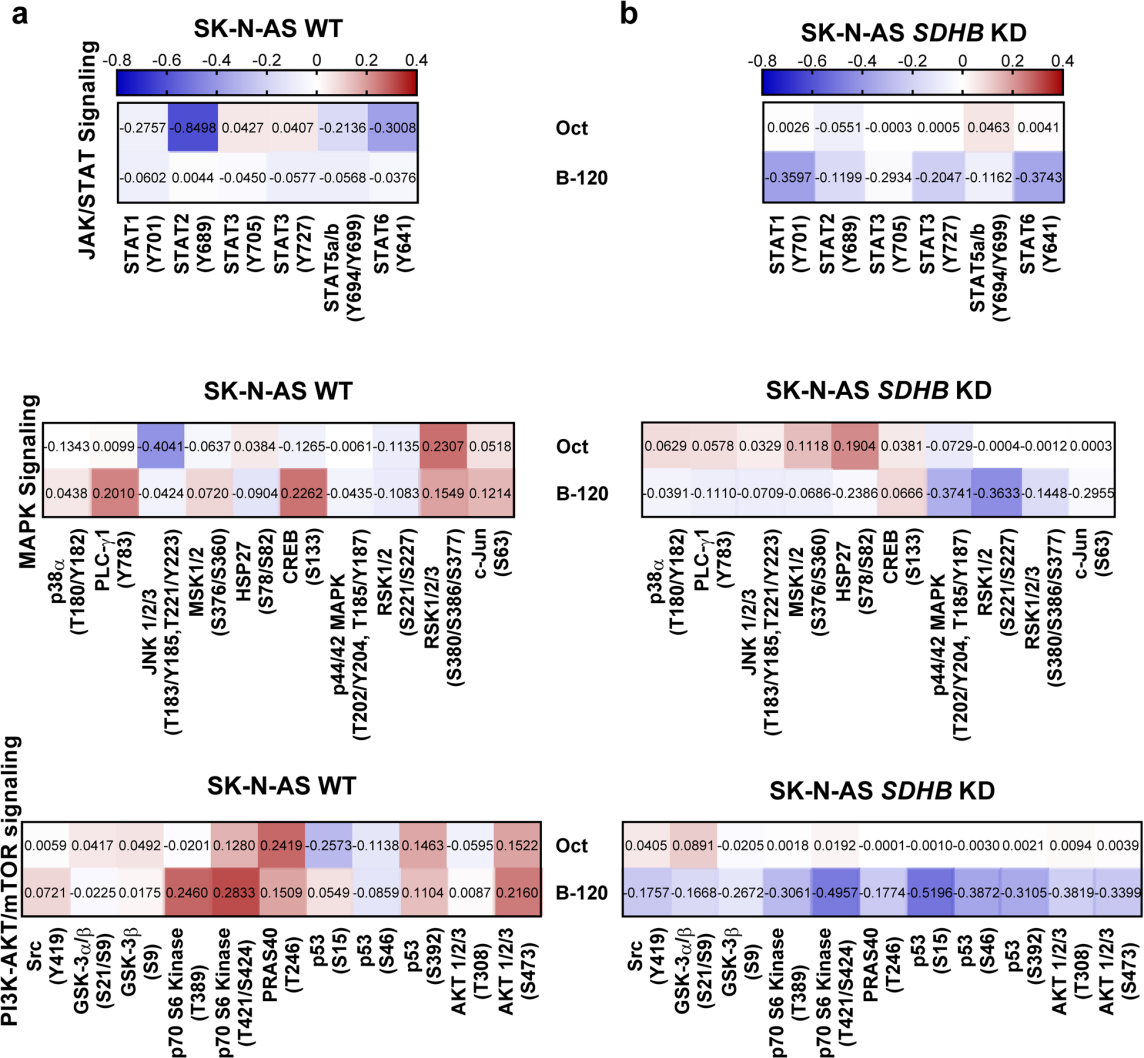
Modification of phosphorylation levels of key proteins of cell growth, survival, and proliferation pathways in response to octreotide and BIM-23120. **a**, **b** Phosphorylation level variations of the main JAK/STAT (top), MAPK (middle), and PI3K-AKT/mTOR (bottom) signaling proteins in response to octreotide (top row) and BIM-23120 (bottom row) in the WT and *SDHB*-altered lines. Values represent the mean of duplicate spots from a pool of three biological replicates for each condition and cell line

Marked differences in the effects of octreotide and BIM-23120 treatment, and between *SDHB*-silenced and WT SK-N-AS cells, prompted us to explore the underlying signaling pathways. Phosphoarray analysis revealed significant compound- and cell line-specific changes in MAPK, PI3K-AKT/mTOR and JAK/STAT pathways (Fig. 6, Fig. S4a and b). Specifically, in WT cells, octreotide reduced proliferation signaling, particularly via JAK/STAT, and, to a lesser extent, MAPK signaling. BIM-23120 treatment had moderate or even stimulatory effects, especially on MAPK and PI3K-AKT/mTOR pathways (Fig. 6a). Conversely, in *SDHB*-silenced cells, BIM-23120 triggered a consistent reduction in phosphorylation levels across all three signaling pathways, whereas octreotide had minimal impact (Fig. 6b).

Hence, these results reinforce the dissimilar responses caused by both compounds in each cell line, as well as the different actions exerted by each compound in both cell lines (Fig. 6a and b). To further examine these results, we selected three core proteins of these pathways that showed relevant changes in some of the treatments: AKT, p44/42 MAPK1/2, and GSK-3β (Fig. S4c and d). Western blot demonstrated that in the WT cell line both compounds exerted comparable significant reductions in two of the proteins (p44/42 MAPK1/2 and GSK-3β). In contrast, in *SDHB-*silenced cells, the actions of the two compounds were more dissimilar, since octreotide provoked a dephosphorylation in p44/42 MAPK1/2 (which did not fully reproduce that found in the phosphoarray), but BIM-23120 did exert more significant inhibitions than octreotide on both AKT and GSK-3β.

In summary, these data indicate that BIM-23120 and octreotide elicit distinct, compound- and genotype-specific modulation of key signaling pathways in SK-N-AS cells. BIM-23120 produced more pronounced inhibition of MAPK, PI3K-AKT/mTOR, and JAK/STAT signaling in *SDHB*-deficient cells, suggesting that receptor selectivity and tumor genotype may shape downstream pathway responses.

### The antitumor effects of BIM-23120 in SK-N-AS *SDHB* KD appear to be dependent on SST_2_

BIM-23120 exhibited a robust effect on *SDHB*-deficient cells, apparently driven by alterations in the phosphorylation status of key signaling pathways involved in cell growth, survival, and proliferation. However, the association between these effects and SST_2_ activity remained uncertain, beyond the high selectivity of this compound for this receptor. To clarify this, we silenced *SSTR2* using a specific siRNA and re-evaluated the parameters previously affected by BIM-23120, comparing the drug’s action under receptor modulation with the baseline (scramble) condition.

**Fig. 7:**
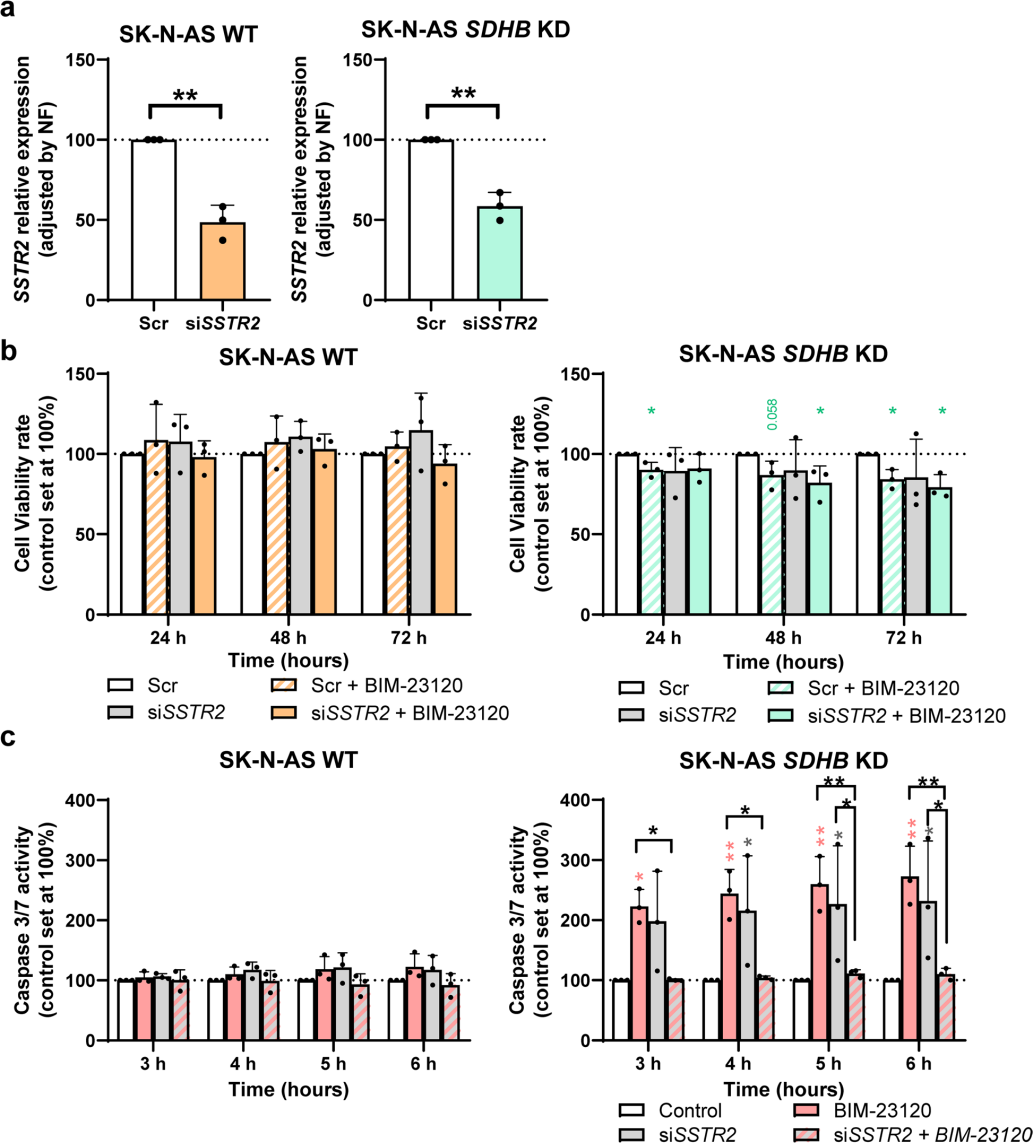
The antitumor effects of BIM-23120 are highly dependent on SST_2_. **a** Validation of *SSTR2* silencing in SK-N-AS WT (left, orange) and SK-N-AS *SDHB* KD (right, green) cell lines (*n* = 3). **b** Changes in cell viability at 24, 48, and 72 h in SK-N-AS WT (left, orange) and SK-N-AS *SDHB* KD (right, green) cells in response to BIM-23120 treatment, *SSTR2* silencing, and their combination (*n* = 3). **c** Apoptosis assay in SK-N-AS WT (left) and SK-N-AS *SDHB* KD (right) cells following BIM-23120 treatment, *SSTR2* silencing, or the combination of both (*n* = 3). Colored asterisks indicate statistically significant differences compared with the control group. Black asterisks above brackets denote statistically significant differences between the indicated groups (* *p* < 0.05, ** *p* < 0.01)

Approximately, 50% gene silencing was achieved in both cells (SK-N-AS WT and *SDHB* KD) (Fig. 7a). In WT cells, both BIM-23120 treatment, *SSTR2* silencing or their combination similarly resulted in an apparent lack of effect on cell viability (Fig. 7b). In line with this, a comparable lack of changes was observed for apoptosis by BIM-23120 incubation, *SSTR2* silencing or their combination (Fig. 7c).

In contrast, in *SDHB* KD cells, BIM-23120 led to a modest but significant reduction in cell viability. This effect was only slightly precluded by *SSTR2* silencing at 24 h, but not at longer incubation times, whereas *SSTR2* silencing alone resulted in small, variable non-significant changes (Fig. 7b). Interestingly, measurement of apoptosis revealed that both BIM-23120 treatment and *SSTR2* silencing promoted a similar, long-lasting significant increase in apoptotic activity. In *SSTR2* silenced cells, however, BIM-23120 treatment was unable to alter apoptotic activity, and the apoptosis-inducing action of *SSTR2* silencing itself also seemed to disappear (Fig. 7c).

These results collectively indicate that the pro-apoptotic and antiproliferative effects of BIM-23120 in *SDHB*-deficient SK-N-AS cells are likely dependent on SST_2_. Nevertheless, residual effects observed in its absence suggest that additional SST_2_-independent mechanisms involving other receptors may also contribute.

### SST_2_ exhibits distinct internalization dynamics in both cellular models under basal conditions and upon treatment

**Fig. 8:**
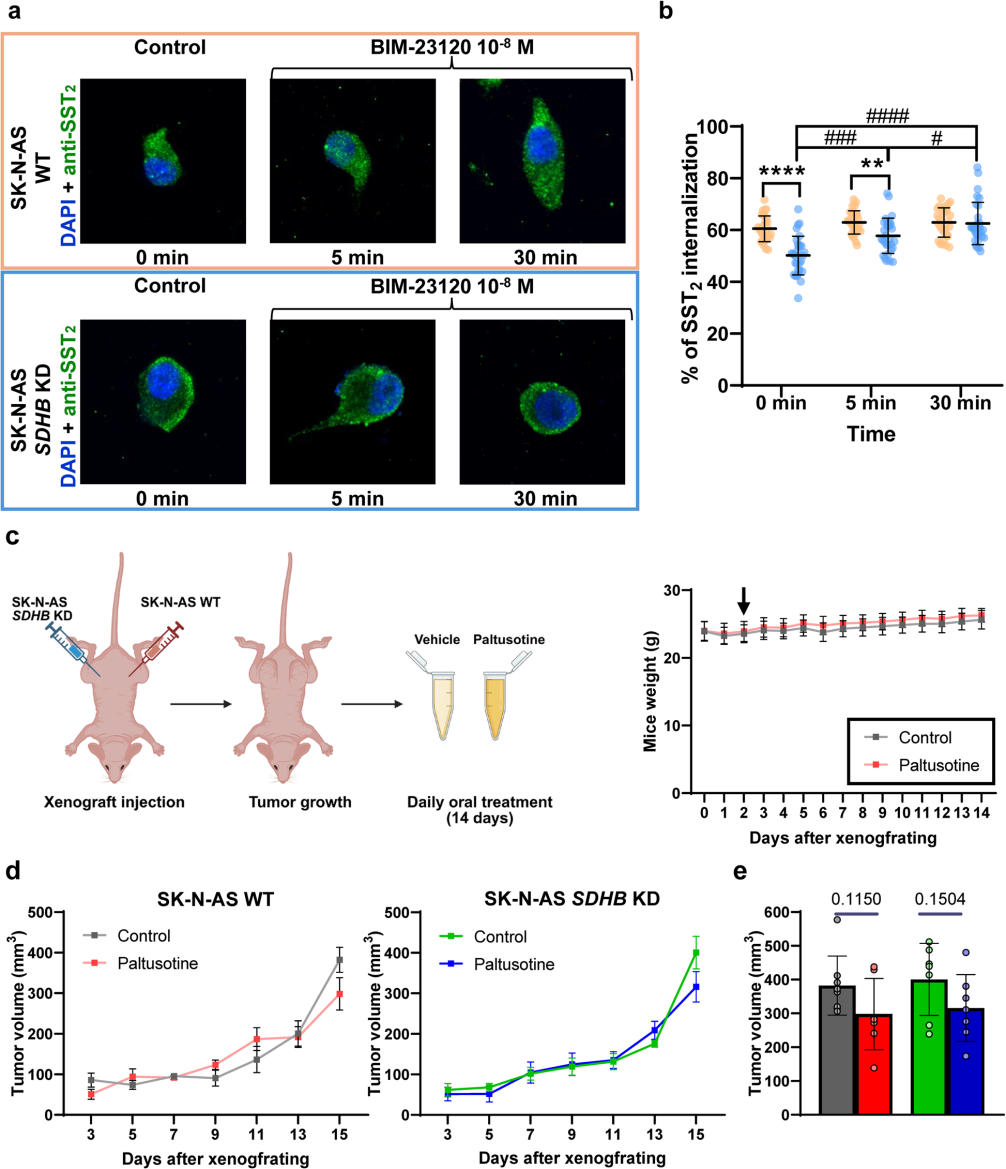
SST_2_ localization and intracellular trafficking in response to BIM-23120, and in vivo response to paltusotine in SK-N-AS WT- and SK-N-AS *SDHB* KD-derived xenografts. **a** Representative immunofluorescence experiment showing subcellular localization of SST_2_ (green) in SK-N-AS WT (orange) and SK-N-AS *SDHB* KD (blue) stimulated or not with 10^–8^ M BIM-23120 for the indicated times. **b** Percentage of SST_2_ internalization (*n* = 30) in SK-N-AS WT (orange) and SK-N-AS *SDHB* KD (blue) cells at 0, 5, and 30 min. **c** Schematic representation of the generation of the preclinical PPGL xenograft model by subcutaneous inoculation of SK-N-AS WT and SK-N-AS *SDHB* KD cells (*n* = 15; left panel), and mean body weight profiles for control (gray) and paltusotine-treated (red) groups throughout the treatment period (right panel). **d** Tumor growth in SK-N-AS WT-derived tumors treated with paltusotine (red) versus control (dark gray) in the left panel, and in SK-N-AS *SDHB* KD-derived tumors treated with paltusotine (dark blue) versus control (dark green) in the right panel. **e** Comparison of tumor size at treatment completion. Unpaired t test was performed to assess statistical analysis between groups. Tumor volume is expressed as mm^3^ and was measured in all the mice every 2 days using a caliper. Black asterisks above brackets denote statistically significant differences between the two cell lines (** *p* < 0.01, **** *p* < 0.0001). Hash symbols above brackets indicate statistically significant differences between time points within the same cell line (# *p* < 0.05, ### *p* < 0.001, #### *p* < 0.0001)

The divergent behavior of the two cell lines regarding SST_2_ signaling was particularly striking, considering that they differ only in the silencing of *SDHB*. Therefore, we sought to characterize the basal localization of this receptor and its dynamic response to treatment. Immunofluorescence (IF) analyses revealed that, under basal culture conditions, *SDHB* KD cells displayed a higher membrane-associated fraction of SST_2_ than WT cells, in which approximately 60% of the receptor was localized intracellularly (Fig. 8a and b). Furthermore, upon BIM-23120 treatment, a progressive receptor internalization was observed in *SDHB* KD cells, with the intracellular fraction increasing from ~ 50% under basal conditions to 58% at 5 min and 62.5% at 30 min. In contrast, WT cells showed minimal changes in receptor distribution, with only a ~ 3% increase in intracellular SST_2_ levels over the same period. These observations suggest that ligand-induced SST_2_ trafficking is impaired in WT cells, whereas *SDHB* silencing seems to enhance receptor membrane localization and its intracellular dynamics in response to ligand activation.

### The complexity of the somatostatin system necessitates the development of specific molecules to facilitate translation of in vitro findings

Given the antitumor potential demonstrated by BIM-23120, we sought to validate these results in vivo using xenograft mouse models. However, due to the discontinuation of BIM-23120, we acquired paltusotine, another selective SST_2_ agonist currently being tested in advanced clinical trials.

First, we tested paltusotine effect in our in vitro models, where a dose–response experiment was performed (Fig. S5a). Paltusotine significantly reduced viability only in WT SK-N-AS cells after 72 h at 10^–8^ and 10^–9^ M. In contrast, the antiproliferative effect in *SDHB*-silenced SK-N-AS cells was less pronounced, with a modest but significant reduction observed at 24 h for 10^–9^ M and at 72 h for 10^–7^ M. PGL1 was unaffected at all concentrations and time points, whereas PGL7 exhibited a mild yet significant decrease in viability at 48 h (10^–7^ M). Based on these results, the 10^–7^ M dose was selected for subsequent experiments, as it reproduced the inhibitory effects observed with BIM-23120 in *SDHB*-silenced SK-N-AS cells.

At this concentration, clonogenic capacity was appreciably reduced in WT SK-N-AS cells, although the decrease did not reach statistical significance, whereas it was significantly decreased in PGL1 cells (~ 50% reduction) (Fig. S5b). Conversely, *SDHB*-silenced SK-N-AS and PGL7 cells showed no appreciable changes in colony-forming ability. Migration assays revealed no effect in any model (Fig. S5c).

After this, xenograft models were established using both SK-N-AS cell types to evaluate in vivo effects on tumor growth. After 14 days of treatment, we observed the formation of 26 tumors out of 32 possible sites (81.25%), with a similar distribution between WT (*n* = 12) and *SDHB*-silenced (*n* = 14) cells. Tumors displayed a generally loose and poorly consolidated appearance in both groups; however, *SDHB* KD-derived tumors showed a markedly higher presence of blood vessels. Notably, all animals remained healthy throughout the experiment and exhibited no significant weight loss or fluctuations (Fig. 8c).

Oral administration of paltusotine did not produce an overt, statistically significant effect on tumor growth in either model. However, a slight, non-significant reduction in tumor size was appreciable at the end of the treatment period (Fig. 8d and e).

Overall, these findings suggest that, under the conditions tested, paltusotine does not significantly impair tumor establishment or progression in this xenograft model.

## Discussion

The high metastatic potential of PPGL, coupled with the limited availability of effective therapies, has driven extensive molecular investigations aimed at improving their diagnosis and management. In recent years, significant progress has been made in elucidating the molecular landscape of these tumors [23–25] and in diagnostic methods, including molecular approaches and advanced imaging techniques [26, 27]. Therapeutic strategies such as PRRT have also advanced, although their clinical benefit remains restricted to certain patient populations [28, 29].

Nevertheless, the lack of correspondence between the robust SSTs expression in PPGL and the limited clinical evaluation of SSAs remains unclear. To address this, we analyzed SSTs expression across a large international cohort and assessed SSAs responses in cell models. Our findings confirm, expand, and refine current knowledge regarding SSTs expression in PPGL, revealing a consistent overexpression of *SSTR2* and *SSTR1* across independent cohorts and cell lines, aligning with early studies [30, 31]. However, we detected a virtual absence of *SSTR4* and *SSTR5* expression consistent with studies reporting a loss of SST_5_ in PPGL [30], although other reports have described elevated SST_5_ levels [31]. In fact, several studies have acknowledged this variability in SSTs expression across PPGL [32].

This heterogeneity was also reflected in our cohorts, where partial discrepancies emerged between qPCR and RNA-seq analyses. While SST_1_ and SST_2_ showed consistent expression across tumor types, notable differences were found when comparing SST_3_, SST_4_ and SST_5_, consistent with prior characterizations in PCC and PGL [16]. These discrepancies are likely attributable, at least in part, to technical factors. Although RNA-seq provides a comprehensive transcriptomic overview, it has limited sensitivity for low-abundance transcripts, particularly when sequence depth is modest or when highly expressed genes dominate the library. In contrast, qPCR is a targeted method with higher sensitivity, allowing detection of transcripts that may be underrepresented in RNA-seq. Previous studies support these limitations, showing that low-abundance and short isoforms are prone to under-detection compared with qPCR [33, 34], while isoform quantification methods more accurately infer highly expressed isoforms [35].

Beyond technical considerations, our findings are also corroborated by alternative experimental approaches, such as Immunohistochemistry (IHC), which show a strong membrane SST_2_ staining in PPGL. This endorses SST_2_ as a suitable diagnostic and/or therapeutic target [32, 36]. Interestingly, at variance with other tumor types, SST_2_ IHC positivity has been proposed as a potential independent marker of metastatic behavior in PPGL, as supported by association with metastatic disease regardless of germline *SDHB* mutations or tumor size [22]. SST_2_ expression also appeared to be related to *SDHB* mutations and higher tumor grade [22, 37], further reinforcing our observation of a heterogeneous, distinct expression profile for SST_2_ signaling molecules across PPGL molecular clusters. While it would have been ideal to confirm the expression profile of the SSTR genes by evaluating the protein levels of SST_1_-SST_5_, sample limitations and technical constraints precluded this possibility. This notwithstanding, the abundant previous evidence derived from immunocytochemistry studies closely corroborates our findings, where SST_2_ unequivocally stands as the most relevant receptor in PPGL.

Our study aimed to understand the limited efficacy of cold SSAs in PPGL despite consistent SSTs overexpression, and to identify potential therapeutic vulnerabilities. To circumvent the scarcity of proper human models [38, 39], we used neuroblastoma-derived cell models, which share a common embryonic origin and mirrored the SSTs expression seen in tumor samples. Native peptides (somatostatin, cortistatin) or clinically used SSAs (octreotide, pasireotide) had minimal antitumor effects, recapitulating the limited clinical response in patients. In contrast, selective SST agonists elicited clear antiproliferative and pro-apoptotic effects that were only evident in *SDHB*-silenced cells, suggesting a subtype-specific vulnerability. These differential responses may be explained by the higher receptor specificity and distinct affinity profiles of the selective agonists compared to the broader binding of somatostatin and SSAs [40]. In this context, treatment of this cellular subtype following *SSTR2* silencing revealed that the antitumor effects triggered by BIM-23120 appear to be likely *SSTR2*-dependent, as no substantial differences were observed between *SSTR2* silencing alone and the combined treatment in terms of cell viability, and a reduction in receptor expression abolished the pro-apoptotic effect of BIM-23120.

Intriguingly, *SSTR2* silencing itself seemed to exert antitumor effects in *SDHB*-deficient SK-N-AS cells, where it increased apoptosis. This may contrast with the general antitumoral role associated to this receptor in other tumors [13]. However, *SSTR2* depletion in Small-Cell Lung Cancer (SCLC) has also been shown to enhance apoptosis and diminish cell viability [41]. In this same vein, the pro-apoptotic effect of *SSTR2* silencing was fully blunted in the presence of BIM-23120, which may result from the reduced SST_2_ levels remaining after silencing or to non-SST_2_ actions of the compound in other SSTs subtypes. In fact, previous studies in SCLC have shown that *SSTR2* silencing increases phosphorylation of STAT6 and AKT [41], proteins that exhibited marked dephosphorylation in SK-N-AS *SDHB* KD cells in response to BIM-23120. The seemingly paradoxical behavior of SST_2_, where both its activation and suppression can elicit antitumor responses, underscores the dynamic nature of this signaling system and highlights the complexity of the functional mechanisms linked to this receptor and its context, which are clearly ligand- and cell type-dependent. SSTs regulate multiple interconnected intracellular cascades, enabling a non-linear response pattern. Consequently, both activation and loss can converge toward similar phenotypic outcomes through distinct molecular mechanisms.

Actually, several studies have reported a remarkable antitumor activity in other NENs of the agonists tested herein, which could even outperform classical SSAs [40, 42, 43]. The somatostatin system’s complex signaling, including receptor interactions and internalization, likely contributes to SSAs resistance. Differential response to SSAs and to specific agonists may lay in the intricate interplay of the somatostatin-SSTs system and its intracellular and signaling dynamics [13]. In fact, treatment responses are not solely determined by their affinity for SSTs but also by receptor interactions and internalization processes, which can lead to the loss of membrane accessibility, preventing therapeutic ligands from exerting their intended effects [44, 45].

In this context, our results showed a higher membrane-associated fraction of SST_2_ in *SDHB* KD cells compared with WT cells, consistent with the increased membrane immunoreactivity in *SDHB*-mutated PPGL [22]. Moreover, WT cells exhibited minimal receptor internalization upon stimulation, suggestive of a refractory receptor state with impaired dynamics and recycling. In contrast, the functionality of ligand-induced receptor trafficking appears restored in *SDHB*-silenced cells, resembling canonical SST_2_ regulation in neuroendocrine models, where dynamic internalization and recycling are essential to maintain sensitivity to SSAs. Indeed, previous studies in NETs have shown that altered SST_2_ trafficking contributes to pharmacological resistance [46, 47]. Thus, it has been proposed to replace the classical SSAs with novel agonists to prevent this loss of activity, since they could have an effect on tumors resistant to SSAs [48–50]. For instance, evidence suggest that refractoriness to SSAs does not imply cross-resistance to agents with similar receptor binding profiles [51]. However, in our models, BIM-23120 monotherapy was generally more effective than its combination with other agents commonly employed in cancer therapy (e.g., everolimus and sunitinib). In several instances, combinatorial treatment attenuated the antitumor effect observed with BIM-23120 alone. This finding highlights the complexity of the somatostatin system, as the signaling pathways through which it exerts its effects are shared with those targeted by other therapeutic agents, potentially leading to mutual interference, as previously described [52]. These results underscore the importance of systematically evaluating drug-drug interactions within this pathway to optimize future combinatorial strategies. In this sense, it is important to place our findings in the context of current PPGL therapeutic strategies. PRRT, particularly with ^177^Lu-DOTATATE, has demonstrated promising efficacy in patients with progressive mPPGL. However, its effectiveness appears reduced in patients harboring SDHx mutations, particularly *SDHB*, who showed shorter progression-free and overall survival compared to sporadic cases [28]. Unlike radiolabeled analogs, BIM-23120 is a cold SSA that acts via receptor modulation rather than radionuclide payload delivery. BIM-23120 exhibited enhanced activity in *SDHB*-altered cell models, suggesting that it may provide therapeutic benefit in patients less responsive to current PRRT or SSAs strategies.

To further explore the dissimilar functional behavior of cells in response to two ligands preferentially targeting the same receptor (albeit with different specificity), we assessed key pathways involved in cell growth, proliferation, and survival by comparing phosphorylation patterns across both cell lines and treatments. SSAs exert antiproliferative effects via SHP-1 protein, which dephosphorylates AKT, PDK1 and GSK-3β [53]. Dephosphorylation activates GSK-3β, triggering activation of p53 and Zac1, and ultimately promoting cell cycle arrest and apoptosis [54]. Our phosphoarray results support the involvement of this pathway in the antitumor effects of SSTs in PPGL [55], aligning with findings in other tumor types [42, 56], including NENs, where SSTs may counteract AKT activation by mTOR inhibitors like everolimus [57–61], although this remains debated [52]. Another avenue by which SST_2_ can mediate their antiproliferative activity is via dephosphorylation of MAPK pathway components, activated by growth factors [55], which would be also consistent with our findings. We hypothesize that these observed dissimilarities may arise from cellular alterations induced by SDHB deficiency, including succinate accumulation, Reactive Oxygen Species (ROS) production, and metabolic reprogramming, all of which over activate the aforementioned pathways [62, 63]. Therefore, this increased basal activation of *SDHB* KD cells compared to WT cells, together with the increased SST_2_ expression [22, 64], may underlie the more pronounced phenotypic effects observed upon BIM-23120 treatment. However, the exact mechanism underlying this selective response remains elusive and warrants further investigation.

On the other hand, the use of the selective SST_2_ agonist paltusotine led us to perform the first experimental characterization of the effects of this compound in PPGL cellular models. In contrast to what was observed for BIM-23120, paltusotine exerted mild antitumor effects on cell viability, showing a slightly greater impact in WT SK-N-AS cells and, particularly, in patient-derived primary cultures. Nevertheless, it produced a pronounced reduction in clonogenic capacity in PGL1 cells. These findings align with a recent study reporting the limited and variable effect of this drug on the viability of NEN cell lines, although a stronger effect was observed in primary cultures [65].

In vivo studies showed that paltusotine did not significantly reduce xenograft tumor growth, despite a possible trend at the end of the experiment. Thus, in this tumor model, paltusotine does not appear to exert a similar tumor size–reducing activity to that reported in human pituitary tumors [66]. Nevertheless, our findings suggest that further studies using improved models that better recapitulate the disease and more potent SST_2_ agonist, in the line of BIM-23120, may help to unequivocally determine whether the promising effects observed in vitro can be reproduced in vivo.

The present findings should be interpreted considering several limitations. The rarity, heterogeneity, genetic complexity, and slow growth of PPGL hinder the development of representative experimental models [38, 39]. In the absence of reliable human PPGL cell lines [39], we employed the rat PCC-derived PC-12 Adh and the human neuroblastoma SK-N-AS cell lines, both widely used and sharing relevant biological features with PPGL [39, 67, 68]. In addition, we evaluated our main findings using two PGL-derived primary cultures (PGL1 and PGL7). Nonetheless, the availability of additional validated human models would enhance translational relevance. Similarly, the limited availability of fresh tumor samples and technical constraints precluded the establishment of Patient-Derived Xenografts (PDX). The discontinuation of BIM-23120 restricted its in vivo evaluation; however, we incorporated the selective SST_2_ agonist paltusotine, currently undergoing Phase III clinical evaluation for acromegaly [66] and NENs (CAREFNDR, NCT07087054), with promising clinical outcomes. Furthermore, while BIM-23120 displayed robust antiproliferative and pro-apoptotic activity, its effects on migration and clonogenicity were modest, and other compounds tested showed limited efficacy. Further studies are therefore warranted to clarify the molecular mechanisms underlying cell line-specific responses and to address challenges related to pharmacokinetics, tumor heterogeneity, and clinical translation. Despite these constraints, we consider that the study is supported by a comprehensive experimental design and multiple independent cohorts, providing a solid framework for future investigations into SST_2_-targeted therapeutic strategies in PPGL.

In sum, our study corroborates, strengthens and expands the evidence that PPGL tumors robustly express SSTs, particularly SST_1_ and SST_2_, which could be exploited, beyond PRRT based therapies, as targets for clinical treatment using new receptor-selective drugs, particularly those directed to SST_2_. Future studies, combining available cell and patient-derived tumor models, should help to clarify the mechanisms that preclude ligand-receptor initiated signaling mechanisms to elicit the typical antitumor effects observed in other NENs, or to exert a subtype-specific action. The described SST_2_-selective analog presented herein represents a valuable candidate to spearhead this needed endeavor to tackle in PPGL, especially in those with *SDHB* alterations and/or metastatic tumors.

## Methods

### Patients and samples

Four independent PPGL cohorts were analyzed, comprising both RNA-seq and qPCR data: (1) qPCR-Marseille (*n* = 49; 41 PCC, 8 PGL); (2) qPCR-CNIO (*n* = 42; 24 PCC, 18 PGL), (3) RNA-seq-CNIO (*n* = 162; 90 PCC, 72 PGL), (4) RNA-seq-TCGA (*n* = 184; 144 PCC, 29 PGL). Clinicopathological data are described in Supp. Table 3. Additionally, TPM expression data for adrenal gland tissue from the GTEx platform were obtained. Further details are provided in Supplementary Methods.

### Cell lines and reagents

In vitro assays were performed in two cell lines derived from human female neuroblastoma, SK-N-AS, (both wild-type, WT, and *SDHB* KD cells, RRID:CVCL_1700), the male rat PCC-derived cell line PC-12 Adh (RRID:CVCL_F659), and two PGL cell lines derived from primary cultures: PGL1 (primary tumor) and PGL7 (metastatic lesion). Cell viability, colony formation, migration, and apoptosis assays in response to different treatments were performed as previously described [69, 70]. These treatments included human somatostatin-14, cortistatin-17, octreotide, pasireotide, subtype-selective SST agonists (BIM-23926, BIM-23120, BIM-355, BIM-23206), everolimus, sunitinib, and paltusotine. Additional details are provided in Supplementary Methods.

### RNA isolation, reverse transcription and analysis of gene expression levels by qPCR

Expression of SSTs was evaluated in two different cohorts of samples (Marseille- and CNIO-qPCR cohorts). RNA isolation, retrotranscription and RNA expression analysis were performed as previously described [69]. Further details are provided in Supplementary Methods.

### Silencing of *SSTR2* in vitro

SK-N-AS WT and *SDHB* KD cell lines were transiently transfected with a siRNA to specifically knockdown the expression of *SSTR2*. Cell viability and apoptosis assays were performed as previously described [70]. Further details are provided in Supplementary Methods.

### Analysis of signaling pathways by human phosphokinase array

Phosphorylation profiling was carried out in WT and *SDHB*-silenced SK-N-AS cells treated with SST_2_ agonists using a human phosphokinase array (Proteome Profiler; R&D Systems, ARY003C, Minneapolis, MN, USA), as previously reported [42]. Detailed procedures are provided in the Supplementary Methods.

### Measurement of key proteins by Western blotting

Protein expression was evaluated by Western blot to validate phosphokinase array findings. Cell lysates were incubated with antibodies against phospho-GSK-3β, phospho-p44/42 MAPK, phospho-AKT, and GAPDH. The detailed protocol is provided in Supplementary Methods.

### Immunofluorescence

SST_2_ basal localization and redistribution in response to BIM-23120 treatment were examined by IF in SK-N-AS WT and *SDHB* KD cells. Detailed procedures are provided in the Supplementary Methods.

### Xenograft model

SK-N-AS WT and *SDHB* KD cells were subcutaneously injected to generate xenograft models in BALB/cAnNRj-Foxn1nu mice (Janvier Labs, Le Genest-Saint-Isle, France; *n* = 16 mice). The detailed protocol is provided in Supplementary Methods.

### Statistical analysis

Transcriptomic data from PPGL cohorts, as results from in vitro and in vivo experiments, were analysed using MetaboAnalyst 6.0 and GraphPad Prism v8.0. Detail methods of data analysis are provided in Supplementary Methods.

## Supplementary Information

Supplementary Material 1.

## Data Availability

All data supporting the findings of this study are available within the paper and its Supplementary Information.
