# Supplementary material for: Functional profiling of somatostatin receptors identifies somatostatin receptor subtype 2 as a vulnerability in Succinate Dehydrogenase SDHB-deficient pheochromocytomas and paragangliomas
Source: Mol Biomed. 2026 Apr 3;7:44. doi: 10.1186/s43556-026-00440-5 (PMC13046876; doi:10.1186/s43556-026-00440-5)
Supplement: Supplementary file 1 — Supplementary Material 1. [file 43556_2026_440_MOESM1_ESM.docx]

**Functional profiling of somatostatin receptors identifies Somatostatin Receptor Subtype 2 as a vulnerability in Succinate Dehydrogenase *SDHB*-deficient pheochromocytomas and paragangliomas**

Víctor García-Vioque^1,2,3^†, Sergio Pedraza-Arevalo^1,2,3^†, María Trinidad Moreno-Montilla^1,2,3^, Esther Rivero-Cortés^1,2,3^, Ricardo Blázquez-Encinas^1,2,3^, Federica Mangili^1,2,3,4^, Ester Arroba^5^, Aura D. Herrera‑Martínez^2,6^, Michael D. Culler^7^, María Ángeles Gálvez-Moreno^2,6^, Anne Barlier^8^, Luisa María Botella^9^, Mercedes Robledo^5,10^*, Justo P. Castaño^1,2,3,11^*, Alejandro Ibáñez-Costa^1,2,3^*.

**Affiliations**

1. Department of Cell Biology, Physiology and Immunology, University of Cordoba, Cordoba, Spain.
2. Maimonides Biomedical Research Institute of Cordoba (IMIBIC), Cordoba, Spain.
3. Reina Sofia University Hospital (HURS), Cordoba, Spain.
4. SC Endocrinology, Fondazione IRCCS Ca' Granda Ospedale Maggiore Policlinico di Milano, Milan, Italy.
5. Hereditary Endocrine Cancer Group, Human Cancer Genetics Program, Spanish National Cancer Research Centre (CNIO), Madrid, Spain.
6. Department of Endocrinology and Nutrition, Reina Sofia University Hospital, Cordoba, Spain.
7. IPSEN Bioscience, Cambridge, Massachusetts, USA, current address: mdculler@comcast.net.
8. Aix Marseille Univ, APHM, INSERM, MMG (U1251), La Timone University Hospital, Laboratory of Molecular Biology GEnOPé, BIOGENOPOLE, Marseille, France.
9. Centro de Investigaciones Biológicas Margarita Salas | CSIC, Madrid, Spain.
10. Centro de Investigación Biomédica en Red de Enfermedades Raras, Madrid, Spain.
11. CIBER Physiopathology of Obesity and Nutrition (CIBERobn), Cordoba, Spain.

**Corresponding authors:** A. Ibáñez-Costa, PhD, email: [b12ibcoa@uco.es](mailto:b12ibcoa@uco.es), telephone: (+34) 957 21 37 38, ORCID: 0000-0003-4649-0095; J.P. Castaño, PhD, email: [justo@uco.es](mailto:justo@uco.es), telephone: (+34) 957 21 37 43, ORCID: 0000-0002-3145-7287, Avenida Menéndez Pidal s/n, IMIBIC Building, 14004, Córdoba, Spain; or M. Robledo, PhD, email: [mrobledo@cnio.es](mailto:mrobledo@cnio.es), ORCID: 0000-0001-6256-5902, Calle de Melchor Fernández Almagro, 3, Fuencarral-El Pardo, 28029, Madrid, Spain.

† Víctor García-Vioque and Sergio Pedraza-Arevalo contributed equally to this work.

**Supplementary Methods**

**Patients and samples**

This study included samples from four independent cohorts, comprising both RNA-seq and qPCR data. Regarding qPCR data, the Marseille cohort consisted of 49 tumor samples (41 PCC and 8 PGL). PPGL samples were collected after surgery with informed written consent from patients, in accordance with ethical protocols approved by the Ethics Committee from Assistance Publique Hopitaux de Marseille and collected after surgery in tumor bank (CODECOH DC 2019-3568, AP-HM tumor bank AC 2013-1786).

The CNIO qPCR cohort comprised 42 tumor samples (24 PCC and 18 PGL). PPGL tumor samples were obtained from patients with informed written consent for this purpose, in accordance with institutional ethical-approved protocols. The protocol was approved by the Ethics Committee of Hospital Universitario 12 de Octubre (15/024, Madrid, Spain); and then gathered at CNIO (Madrid, Spain).

RNA-seq analyses from CNIO series, consisting of 162 PPGL samples (90 PCC and 72 PGL), were performed as previously described [1]. Briefly, HTSeq-counts for the CNIO series were generated from FASTQ files obtained from the TruSeq and QuantSeq platforms using Nextpresso and the BlueBee® Genomics Platform (Lexogen, 090-094), respectively. Libraries prepared with Unique Molecular Identifiers (UMIs) were processed using the QuantSeq FWD-UMI Data Analysis Pipeline. Sequencing reads were aligned to the GRCh37 reference genome using TopHat v2.1.1 [2] and STAR v2.5.2a [3], corresponding to the respective platforms. RNA-seq fastq files generated are deposited in the European Genome-Phenome Archive (EGA) under the accession EGAS00001006043 and EGAS00001006044. Request for data access will be referred directly to the Data Access Committee (DAC) of the CNIO ([mrobledo@cnio.es](mailto:mrobledo@cnio.es)). The access will be granted for health/medical/biomedical purposes and according to good practice recommendations.

Additionally, we analyzed bulk RNA-seq data from 184 PPGL samples (144 PCC and 29 PGL) obtained from The Cancer Genome Atlas (TCGA) (<https://portal.gdc.cancer.gov/>), from which samples catalogued as cortical admixture were excluded for the molecular cluster analyses. Clinical data and RSEM normalized mRNA expression were downloaded from the cBioPortal portal for further analysis [4]. In addition, we downloaded TPM expression data for adrenal gland tissue from the GTEx platform [5], to compare SSTRs expression levels with those observed in normal tissue. To enable this comparison, batch effects arising from differences in analytical methods, sequencing platforms, and cohort origins were corrected using *ComBat* [6] from R/Bioconductor package *sva* [7] v3.46.0 on log_2_-transformed values.

All experiments were conducted in accordance with the ethical standards of the Helsinki Declaration and with the approval of the University of Cordoba/IMIBIC and the Ethics Committees from the participating hospitals/universities (protocol code PI19/01603, v1 - 13/03/2019). In all cases, the study was undertaken after informed consent was obtained from each patient. Clinicopathological data of all cohorts analyzed in this study are described in Supp. Table 3.

**Cell lines and reagents**

Cells derived from human female neuroblastoma, SK-N-AS, (both wild-type, WT, and *SDHB* KD cells, [RRID:CVCL_1700](https://rrid.site/data/record/SCR_013869-1/CVCL_1700/resolver?q=CVCL_1700&l=CVCL_1700&i=rrid:cvcl_1700-0)) were kindly provided by Dr. Massimo Manelli (University of Florence, Florence, Italy) [8], and male rat PCC-derived cell line PC-12 Adh ([RRID:CVCL_F659](https://rrid.site/data/record/SCR_013869-1/CVCL_F659/resolver?q=CVCL_F659&l=CVCL_F659&i=rrid:cvcl_f659-0)) was generously provided by Dr. M. M. Malagon (University of Cordoba, Cordoba, Spain). Mycoplasma testing was performed biweekly to ensure culture integrity by PCR as previously described [9]. The SK-N-AS cell lines were cultured in Dulbecco’s Modified Eagle Medium with 4500 mg/L of glucose (DMEM 4.5 g/L glucose, Corning, Arizona, USA) supplemented with 10% Fetal Bovine Serum (FBS, Sigma-Aldrich, Madrid, Spain), 2 mM L-glutamine (Sigma-Aldrich), 1% antibiotic-antimycotic and G418 (0.2 mg/mL) (Gibco, Madrid, Spain), the latter used to maintain the selection of KD cells. The PC-12 Adh cell line was cultured in DMEM 4.5 g/L glucose supplemented with 10% FBS, 5% Horse Serum (HS, ThermoFisher-Scientific, Waltham, MA, USA), 2 mM L-glutamine and 1% antibiotic-antimycotic. Two cell lines derived from primary cultures were used to test the main findings obtained in the previously described models: PGL1 and PGL7. PGL1 corresponds to a sample derived from a primary pelvic PGL (primary tumor) of a 34-year-old male patient harboring an *SDHB* mutation, whereas PGL7 was derived from a metastatic cervical PGL (metastatic lesion) of a 19-year-old male patient carrying an *SDHB* mutation [heterozygous 5-bp deletion in exon 2 (c.166_170delCCTCA)]. These cells, kindly provided by Dr. Botella, were cultured in RPMI-1640 medium (Cytiva, Barcelona, Spain), supplemented with FBS at 20%, L-glutamine at 1%, antibiotic/antimycotic at 0.2% and MycoZap Plus-CL at 0.2% (Lonza, Basel, Switzerland). Human somatostatin-14, cortistatin-17 and octreotide were purchased from Polypeptide Group (Neuhofstrasse, Switzerland), while pasireotide was kindly provided by Herbert A. Schmid [Novartis Pharmaceuticals Corporation (Basel, Switzerland)]. Subtype selective SST_1_, SST_2_, SST_3_ and SST_5_ agonists (BIM-23926, BIM-23120, BIM-355 and BIM-23206, respectively) were generously provided by IPSEN Bioscience, Inc (Cambridge, MA, USA). The affinity of these ligands for each of the SSTs has been summarized in Supp. Table 2 [10–17]. Preliminary dose-response experiments were performed at 10^-10^ M, 10^-8^ M, and 10^-6^ M for all compounds to determine the most appropriate dosage for our models, resulting in the selection of 10^-6^ M for BIM-23206, 10^-8^ M for BIM-23120, and 10^-10^ M for BIM-23926 and BIM-355, based on the doses that produced the most significant and consistent reductions in cell viability and were supported by previous studies [12,13,18–20]. In addition, further experiments were performed using everolimus at a concentration of 10^-9^ M (S1120, Selleck Chemicals, Houston, TX, USA) and sunitinib at a concentration of 10^-7^ M (PZ0012, Sigma-Aldrich). The selection of these concentrations was based on our previous experience in other NET cell lines and published studies involving the aforementioned cell lines [21,22]. Additionally, in vitro and in vivo experiments were performed using paltusotine (Cayman Chemical, MI, USA), a nonpeptide SST_2_ agonist. For the in vitro assays, dose-response experiments were conducted to determine the most appropriate concentration for our models, resulting in the selection of 10^‑7^ M. For the in vivo experiments, a dose of 10 mg kg^-1^ day^-1^ was chosen, as it represents the lowest effective dose reported in the literature [23].

**RNA isolation, reverse transcription and analysis of gene expression levels by qPCR**

The samples were processed for total RNA recovery using AllPrep DNA/RNA/Protein Mini Kit (Qiagen, Madrid, Spain) following the manufacturer’s protocol and subsequently treated with DNase (Promega, Barcelona, Spain). Total RNA from the cell lines was isolated using TRIzol Reagent (Invitrogen, Barcelona, Spain) according to the manufacturer's instructions and treated with DNase. Subsequently, the amount and purity of the isolated RNA was determined using the Nanodrop One Microvolume UV-Vis Spectrophotometer (ThermoFisher-Scientific). One μg of RNA was retro-transcribed to cDNA employing random primers with the RevertAid RT Reverse Transcription Kit (ThermoFisher-Scientific, K1691) in a final volume of 20 μL. Gene expression levels were measured by qPCR using 50 ng of cDNA and Brilliant III SYBR Green Master Mix (Stratagene, La Jolla, CA, USA) in the Stratagene Mx3000p system (Agilent Technologies, Santa Clara, CA, USA), as previously described by our group [24]. We adjusted RNA levels with three housekeeping genes (*ACTB*, *GAPDH* and *HPRT1*), using geNorm software [25], whose levels were stable and without differences among sample groups.

**Silencing of *SSTR2* in vitro**

SK-N-AS WT and *SDHB* KD cell lines were transiently transfected with a siRNA to specifically knockdown the expression of *SSTR2* (ThermoFisher Scientific, Cat #4392421, Waltham, MA, USA). As a control, cells were transfected with Silencer Select Negative Control siRNA (ThermoFisher-Scientific). To this end, 250,000 cells were seeded in 6-well plates and transfected with 75 nM siRNA using lipofectamine RNAiMAX reagent (ThermoFisher-Scientific) at 37 ºC, following manufacturer’s instructions.

**In vitro studies**

**Measurement of cell viability**

Cell viability of PC-12 Adh, WT and *SDHB*-silenced SK-N-AS, PGL1 and PGL7 in response to *SSTR2* silencing or drug administration was assessed using Alamar-Blue (ThermoFisher-Scientific) fluorescent assay, as reported previously [24]. Briefly, cells were seeded in 96-well plates at a density of 5,000 cells/well and serum starved for 12 h. Then, cell viability was assessed at 5% FBS every 24 h using the FlexStation 3 Multi-Mode Microplate reader (Molecular devices, San Jose, CA, USA) until 72 h. Results were expressed as a percentage with respect to vehicle-treated controls, where the vehicle refers to Silencer Select Negative Control siRNA or the solvent used to resuspend each treatment.

**Measurement of colony formation**

To determine the effect of the different treatments on the clonogenic capacity of WT and *SDHB*-silenced SK-N-AS, PGL1, and PGL7 cells, 4,000 cells/well were seeded in 6-well plates after 24 h of treatment. These cells were incubated for 10 days, changing medium every 3 days, then the medium was removed, the colonies were washed with PBS, stained with crystal violet solution (0.5% crystal violet and 6% glutaraldehyde) for 30 min and air dried. Finally, images collected were analyzed using ImageJ v.1.53 (NIH, Bethesda, MD, USA, [RRID:SCR_003070](https://rrid.site/data/record/nlx_144509-1/SCR_003070/resolver?q=SCR_003070&l=SCR_003070&i=rrid:scr_003070)) to determine the number of individual colonies.

**Measurement of cell migration**

The ability of PC-12 Adh, WT and *SDHB*-silenced SK-N-AS, PGL1, and PGL7 cells to migrate after treatment with the above mentioned compounds (24 h) was assessed by wound-healing assay as previously reported [24]. Briefly, 50,000 cells were seeded in 96-well plates and serum-starved for 24 h to achieve cell cycle synchronization and the wound was performed with a 100 μL sterile pipette tip. Subsequently, the wells were washed with PBS and the cells were incubated for 24 and 48 h with medium supplemented without FBS. Wound images were acquired with Moticam 4000 equipment (MoticEurope, Barcelona, Spain) and analyzed with ImageJ software. Wound-healing was compared with the wound area just after wounding.

**Measurement of caspase activity**

WT and *SDHB*-altered SK-N-AS were seeded at a density of 10,000 cells/well in 96-well plates and treated with octreotide and BIM-23120 or transfected with *SSTR2* siRNA. After 48 h, caspase-3/7 enzyme activity was determined using Apo-ONE Homogeneous Caspase-3/7 Assay (Promega) according to the manufacturer's instructions. In brief, caspase-3/7 substrate was diluted 1:100 in their commercial buffer and added to the cells. After 3-6 h of incubation, fluorescence was measured at a wavelength of 499/521 nm with the FlexStation 3 Multi-Mode Microplate reader as previously described [26].

**Analysis of signaling pathways by human phosphokinase array**

As previously reported [16] to test altered signaling pathways in WT and *SDHB*-silenced SK-N-AS treated with SST_2_ agonists, 50,000 cells/well were seeded in 6-well plates and incubated for 15 min with vehicle, octreotide and BIM-23120. Specifically, three biological replicates of each condition (vehicle/treatment; WT/*SDHB* KD) were pooled and 600 μg of cell lysate was loaded onto array membranes to determine the relative phosphorylation levels of 37 critical kinase phosphorylation sites, following the manufacturer's instructions (Proteome Profiler; R&D Systems, ARY003C, Minneapolis, MN, USA). The average signal of each pair of duplicated dots, representing each phosphorylated site, was calculated after subtracting the background values (pixel density) of the negative control dots and normalized to the average values of the positive controls using ImageJ software.

**Measurement of key proteins by Western blotting**

To determine protein levels and validate the results found in the phosphokinase array, cell lysates obtained with the commercial lysis buffer of this kit were separated on Mini-PROTEAN TGX polyacrylamide precast gels (Bio-Rad Laboratories, Madrid, Spain, Cat# 4568126) by SDS-PAGE and transferred to nitrocellulose membranes (Millipore, Burlington, MA, USA, Cat# 1704270). The membranes were then blocked with a solution of 5% non-fat dry milk in Tris-buffered saline containing 0.05% Tween-20 (Sigma-Aldrich, Cat# 93773). The membranes were then incubated with the corresponding primary antibodies, including phospho-GSK-3β (1:1000, Cell Signaling, Danvers, MA, USA; Cat# 9336, [RRID:AB_331405](https://rrid.site/data/record/nif-0000-07730-1/AB_331405/resolver?q=AB_331405&l=AB_331405&i=rrid:ab_331405-96343)), phospho-p44/42 MAPK (1:1000, Cell Signaling, Cat# 4370S, [RRID:AB_2315112](https://rrid.site/data/record/nif-0000-07730-1/AB_2315112/resolver?q=AB_2315112&l=AB_2315112&i=rrid:ab_2315112-2197276)), phospho-AKT (1:1000, Cell Signaling, Cat# 13038S, [RRID:AB_2629447](https://rrid.site/data/record/nif-0000-07730-1/AB_2629447/resolver?q=AB_2629447&l=AB_2629447&i=rrid:ab_2629447-2564903)) and GAPDH (1:2000, ThermoFisher-Scientific, Cat# AM4300, [RRID:AB_2536381](https://rrid.site/data/record/nif-0000-07730-1/AB_2536381/resolver?q=AB_2536381&l=AB_2536381&i=rrid:ab_2536381-2471837)). They were then incubated with secondary anti-rabbit antibody (1:2000, Cell Signaling, Cat# 7074S, [RRID:AB_2099233](https://rrid.site/data/record/nif-0000-07730-1/AB_2099233/resolver?q=AB_2099233&l=AB_2099233&i=rrid:ab_2099233-1241879)) or secondary anti-mouse antibody (1:2000, Cell Signaling, Cat# 7076, [RRID:AB_330924](https://rrid.site/data/record/nif-0000-07730-1/AB_330924/resolver?q=AB_330924&l=AB_330924&i=rrid:ab_330924-738273)) and the proteins were revealed using Clarity Western-ECL Blotting Substrate (Bio-Rad Laboratories). The resulting blots were scanned with an ImageQuant Las 4000 system (GE Healthcare Europe GmbH, Madrid, Spain) and densitometric analysis with ImageJ was performed on the resulting images.

**Immunofluorescence**

SK-N-AS WT and *SDHB* KD cells were seeded on 25-mm coverslips at a density of 50,000 cells/well in 6-well plates and incubated at 37 °C for 24 h. Subsequently, cells were either treated or not with BIM-23120 10^-8^ M for 5 or 30 min. After treatment, cells were fixed with 4% paraformaldehyde (ThermoFisher-Scientific) for 20 min at room temperature, permeabilized with 0.2% PBS-Tween 20, and incubated with blocking solution (1% PBS-BSA) for 30 min at room temperature. Cells were then incubated overnight at 4 °C with an anti-SST_2_ antibody (ThermoFisher-Scientific, Cat# 704011, RRID:AB_2895896). The following day, cells were incubated with an Alexa Fluor 647-conjugated secondary antibody (ThermoFisher-Scientific, Cat# A-21245) for 1 h at room temperature, counterstained with 4′,6-Diamidino-2-Phenylindole (DAPI) (Sigma-Aldrich), and mounted using fluorescence mounting medium (Dako, S3023). Cell preparations were visualized with a Leica Thunder microscope (Leica Microsystems, Barcelona, Spain, RRID:SCR_023794; Microscopy facility, IMIBIC), images were processed using the LAS X software v3.10.1 (Leica Microsystems, RRID:SCR_013673) and analyzed with ImageJ to study SST_2_ cell distribution.

**Xenograft model**

One million SK-N-AS WT and *SDHB* KD cells were injected in each flank of 7-week-old male BALB/cAnNRj-Foxn1nu mice (Janvier Labs, Le Genest-Saint-Isle, France; n = 16 mice), resuspended in 100 µL of Matrigel Matrix (Corning) and DMEM 4.5 g/L glucose supplemented with 10% FBS (1:1 ratio). After three days, when the tumors became palpable, mice were randomly divided into two homogeneous groups based on body weight: a control group, which received a daily oral administration of the vehicle diluted in corn oil (10% vehicle / 90% corn oil) (Sigma-Aldrich) for 14 consecutive days, and a treatment group, which received paltusotine formulated in the same vehicle mixture at a dose of 10 mg per kilogram and day. For dose selection, we chose the lowest effective dose used in previous in vivo studies [23]. Tumor growth was monitored twice per week for 4 weeks by using a digital caliper, and all animals were sacrificed 15 days after the start of treatment. All experimental procedures were carried out following the European Regulations for Animal Care, in accordance with guidelines and regulations, and under the approval of the University of Cordoba Research Ethics Committee.

**Statistical analysis**

First, the data were evaluated for parametric distribution with the Kolmogorov-Smirnov test and expressed as median ± IQR (Interquartile Range) or mean ± SD. Statistical comparisons between groups were performed by unpaired parametric t-test and nonparametric Mann-Whitney U-test, depending on the normality of the data. For groups with three or more variables, one-way ANOVA analysis or Kruskal-Wallis test were performed. For experiments involving multiple treatment groups, statistical significance versus the control group is indicated by an asterisk placed above the corresponding bar and colored according to the group. Significant differences between treatment groups are denoted by black asterisks placed above a bracket connecting the compared groups. PLS-DA (Partial Least Squares-Discriminant Analysis) and VIP (Variable Importance in Prediction) Score analyses were performed in order to pinpoint the most discriminant molecules among the different groups of samples available. These analyses as well as unsupervised hierarchical clustering heatmaps were carried out using MetaboAnalyst 6.0 [27]. At least three different replicates of the in vitro experiments were carried out. Statistical significance was set at * *p* < 0.05; ** *p* < 0.01; *** *p* < 0.001, **** *p* < 0.0001. Statistical analyses were performed using Prism v8.0 (GraphPad, La Jolla, CA, USA, [RRID:SCR_002798](https://rrid.site/data/record/nlx_144509-1/SCR_002798/resolver?q=SCR_002798&l=SCR_002798&i=rrid:scr_002798)).

**Supplementary Tables**

**Supp. Table 1.** List of genes implicated in downstream signaling upon ligand binding to SST_2_.

| **Receptor** | *SSTR2* | |
| --- | --- | --- |
| **Adenylate cyclase pathway (cell death & survival)** | *GNAI1* | *PLAGL1* |
|  | *GNAI2* | *FOS* |
|  | *GNAI3* | *JUN* |
|  | *ADCY1* | *PRKACA* |
|  | *CREB3* |  |
| **Cell cycle** | *GNAI1* | *CDC6* |
|  | *GNAI2* | *RB1* |
|  | *GNAI3* | *RBL1* |
|  | *CCNA* | *ORC1* |
|  | *CCND1* | *E2F1* |
|  | *CCNE* | *E2F4* |
|  | *CDKN1A* | *E2F5* |
|  | *CDK4* | *TFDP1* |
|  | *CDK2* |  |
| **Proliferation (JAK/STAT, MAPK & PI3K-mTOR pathways)** | *GNAI1* | *RAF1* |
|  | *GNAI2* | *MAP2K1* |
|  | *GNAI3* | *MAP2K2* |
|  | *JAK1* | *MAPK1* |
|  | *PTPN2* | *MAPK3* |
|  | *PTPN6* | *PIK3CA* |
|  | *PTPN11* | *PIK3CB* |
|  | *GRB2* | *PIK3CD* |
|  | *SOS* | *AKT* |
|  | *HRAS* | *MTOR* |
| **Antiangiogenic (nitric oxide pathway)** | *NOS1* | *GUCY1A2* |
|  | *NOS2* | *GUCY1B1* |
|  | *NOS3* | *GUCY1B2* |
|  | *GUCY1A1* | *PRKG1* |

**Supp. Table 2.** Affinity of the different ligands used in this study for each of the SSTs.

|  | **IC_50_ (nM)** | | | | |
| --- | --- | --- | --- | --- | --- |
| **Compound** | **SST_1_** | **SST_2_** | **SST_3_** | **SST_4_** | **SST_5_** |
| **Somatostatin-14** [10] | 2.26 | 0.23 | 1.43 | 1.77 | 0.88 |
| **Cortistatin-17** [11] | 7 | 0.6 | 0.6 | 0.5 | 0.4 |
| **Octreotide** [13] | 1140 | 0.56 | 34.5 | 7030 | 7 |
| **Pasireotide** [15] | 9.3 | 1 | 1.5 | >100 | 0.16 |
| **BIM-23120** [12] | 1000 | 0.34 | 412 | 1000 | 213.5 |
| **BIM-23206** [14] | 1152 | 166 | 1000 | 1618 | 2.4 |
| **BIM-355** [16] | 224 | NA | 4 | NA | 5.7 |
| **BIM-23926** [17] | 3.6 | >1000 | >1000 | 833 | 788 |

**Supp. Table 3.** Clinical characteristics of the patients included in every cohort.

| **Cohort** | **Marseille** | | **CNIO** | | **CNIO** | | **TCGA** | |
| --- | --- | --- | --- | --- | --- | --- | --- | --- |
| **Technology** | qPCR | | qPCR | | RNA-seq | | RNA-seq | |
| **Tumor type** | PCC | PGL | PCC | PGL | PCC | PGL | PCC | PGL |
|  | 41 | 8 | 24 | 18 | 90 | 72 | 144 | 29 |
| **Age, mean ± SD** | 49.3 ± 3.3 | 32.4 ±  4.4 | 50.8 ± 9.6 | 41.2 ± 24.17 | 47.8 ± 16.8 | 41 ± 18 | 46.9 ± 1.3 | 49.1 ± 2.9 |
| **Female number (%)** | 21 (51.3) | 4  (50) | 13  (53.8) | 8  (43.8) | 40 (45.5) | 35 (48.6) | 81 (56.3) | 17 (58.6) |
| **Male number (%)** | 20 (48.7) | 4  (50) | 11  (46.2) | 10  (56.2) | 48 (54.5) | 37 (51.4) | 63 (43.7) | 12 (41.4) |
| **Malignant (%)** | NA | NA | NA | NA | 43.3 | 61.1 | 6.9 | 20.7 |
|  | **Molecular cluster** | | | | | | | |
| **Pseudohypoxia** | NA | NA | 2 | 11 | 26 | 54 | 35 | 26 |
| **Kinase signaling** | NA | NA | 6 | 2 | 54 | 17 | 65 | 3 |
| **Wnt-altered** | NA | NA | 1 | 0 | 9 | 0 | 22 | 0 |

PCC: pheochromocytomas; PGL: paragangliomas.

**Supplementary Figures**

**
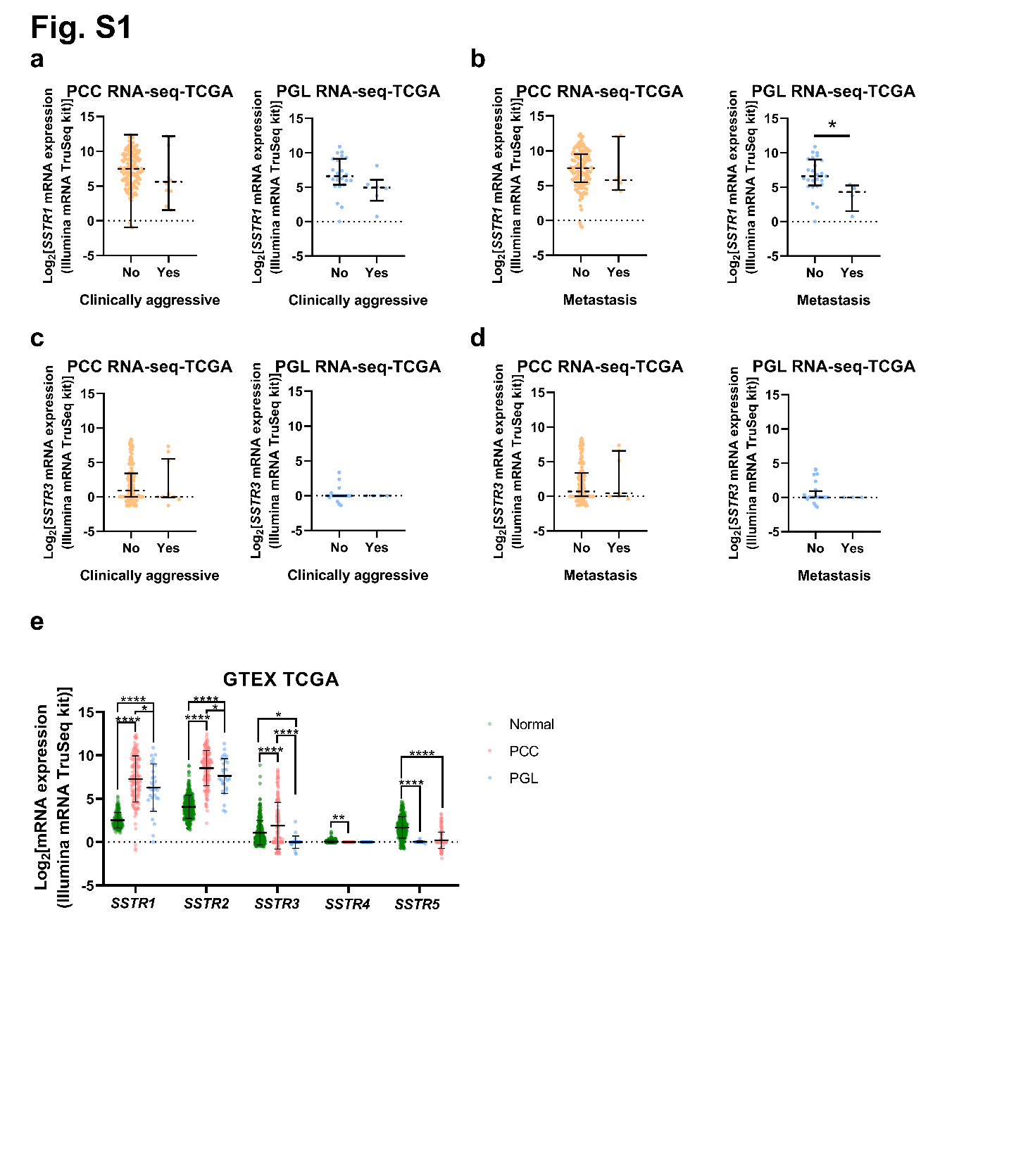
**

**Fig. S1** Association of *SSTR1* and *SSTR3* expression with relevant clinical parameters, and comparison of SSTs expression compared to normal adrenal gland tissue. **a,b** Association of relative *SSTR1* mRNA expression with clinically aggressive behavior and metastasis in PCC (orange) and PGL (blue) in the TCGA cohort. **c,d** Association of relative *SSTR3* mRNA expression with clinically aggressive behavior and metastasis in PCC (orange) and PGL (blue) in the TCGA cohort. Median and interquartile range are represented. **e** Relative mRNA expression of SSTs in PCC and PGL in the TCGA RNA-seq cohort and in normal adrenal gland tissue in the GTEX cohort. Black asterisks above brackets denote statistically significant differences between the indicated groups (* *p* < 0.05, ** *p* < 0.01, *** *p* < 0.001, **** *p* < 0.0001).


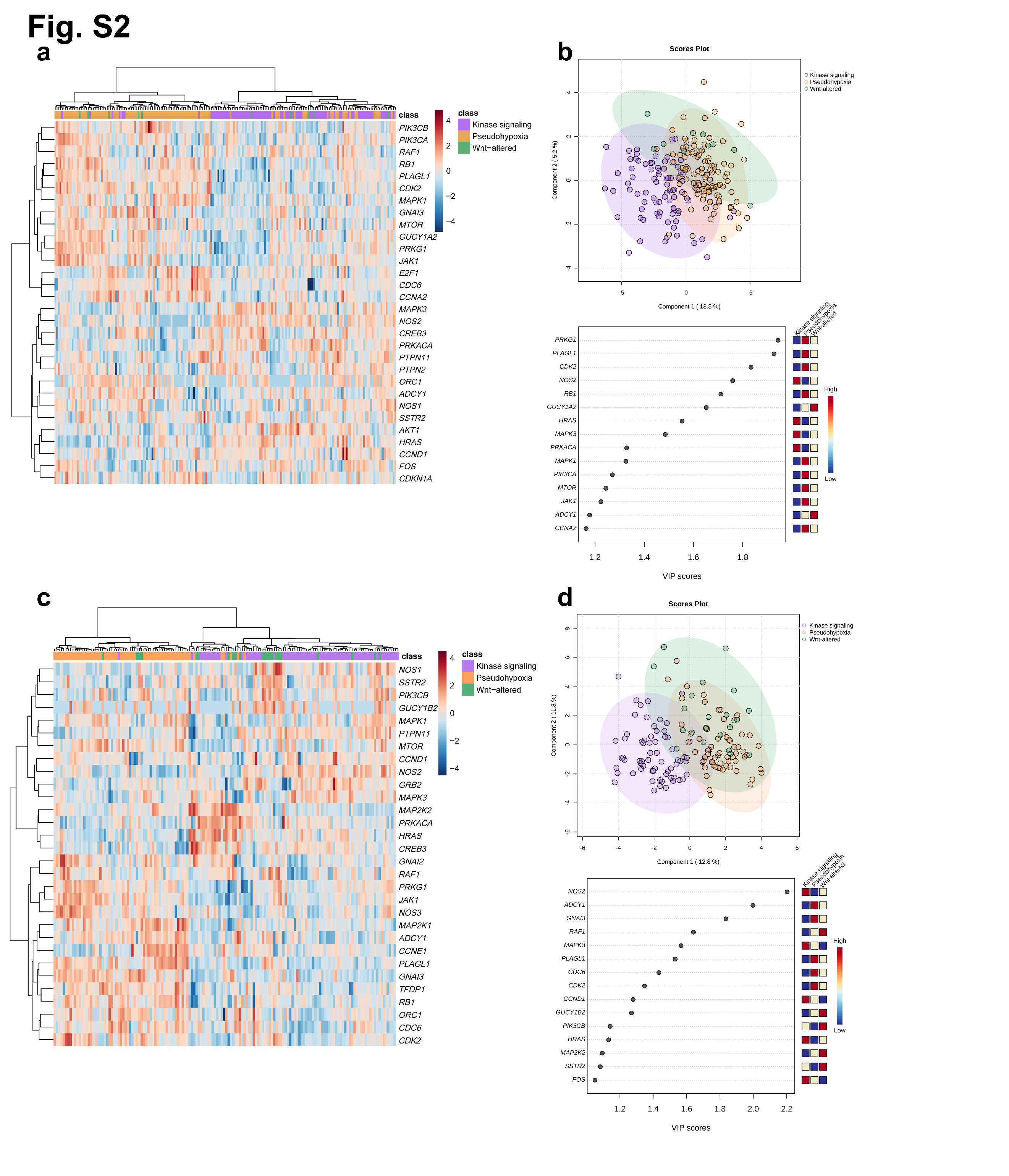


**Fig. S2** Clustering of key genes involved in *SSTR2* signaling across molecular subtypes of PPGL. **a,c** Unsupervised hierarchical heatmap of the expression levels of the top 30 genes involved in SST_2_ signaling that contribute most to the discrimination between the kinase signaling cluster (purple) and the pseudohypoxia cluster (orange) in the CNIO cohort and the TCGA cohort. **b,d** PLS-DA and VIP score of the expression levels of genes involved in SST_2_ signaling in the CNIO cohort and the TCGA cohort. Kinase signaling cluster samples are shown in purple, pseudohypoxia cluster samples are shown in orange, and Wnt-altered cluster samples are shown in green.


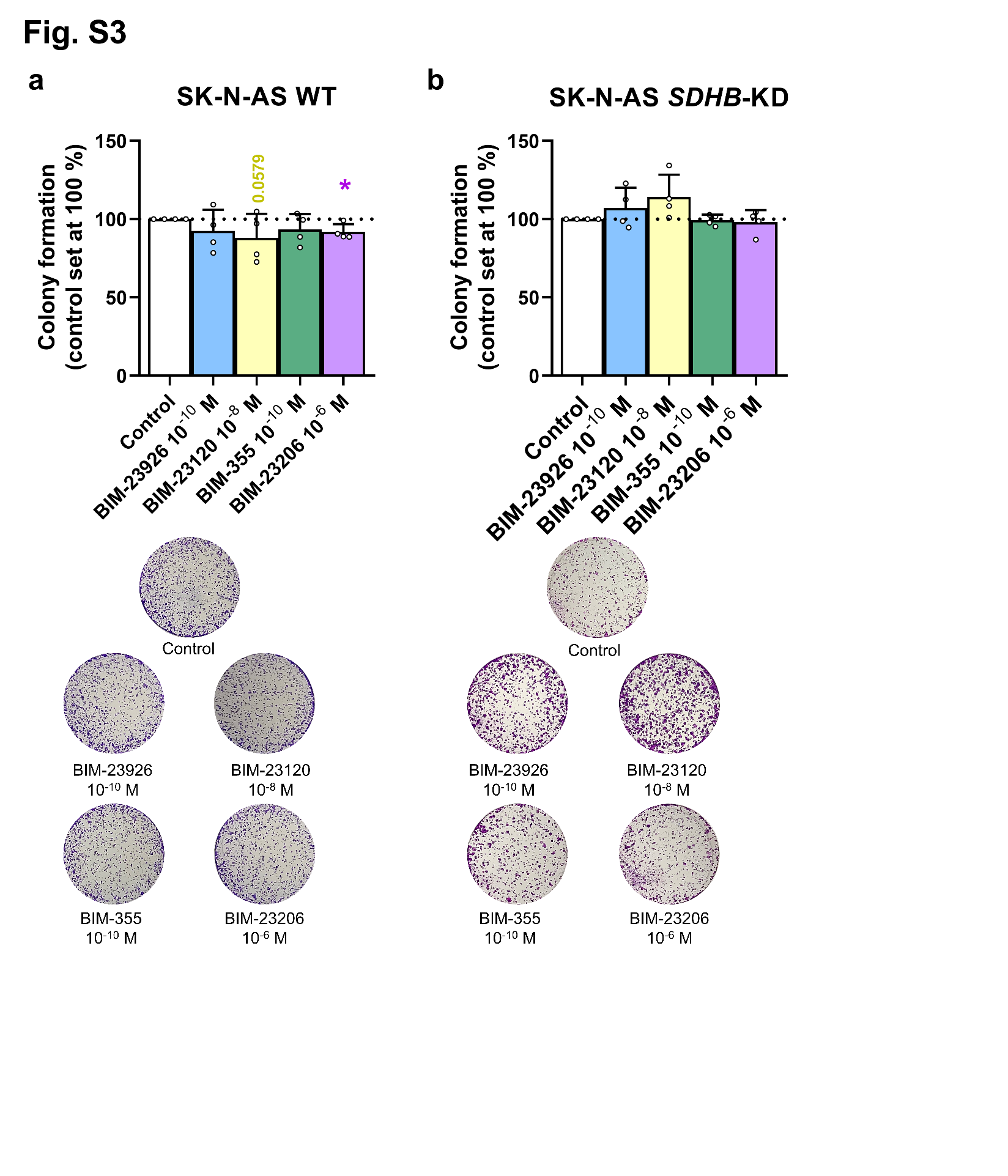


**Fig. S3** Colony-forming capacity of PPGL cell lines in response to specific agonists. **a,b** Evaluation and representative images of colony formation in SK-N-AS WT and SK-N-AS *SDHB* KD responding to therapy with the selective agonists for each SST (n = 4). Colored asterisks indicate statistically significant differences compared with the control group (* *p* < 0.05).


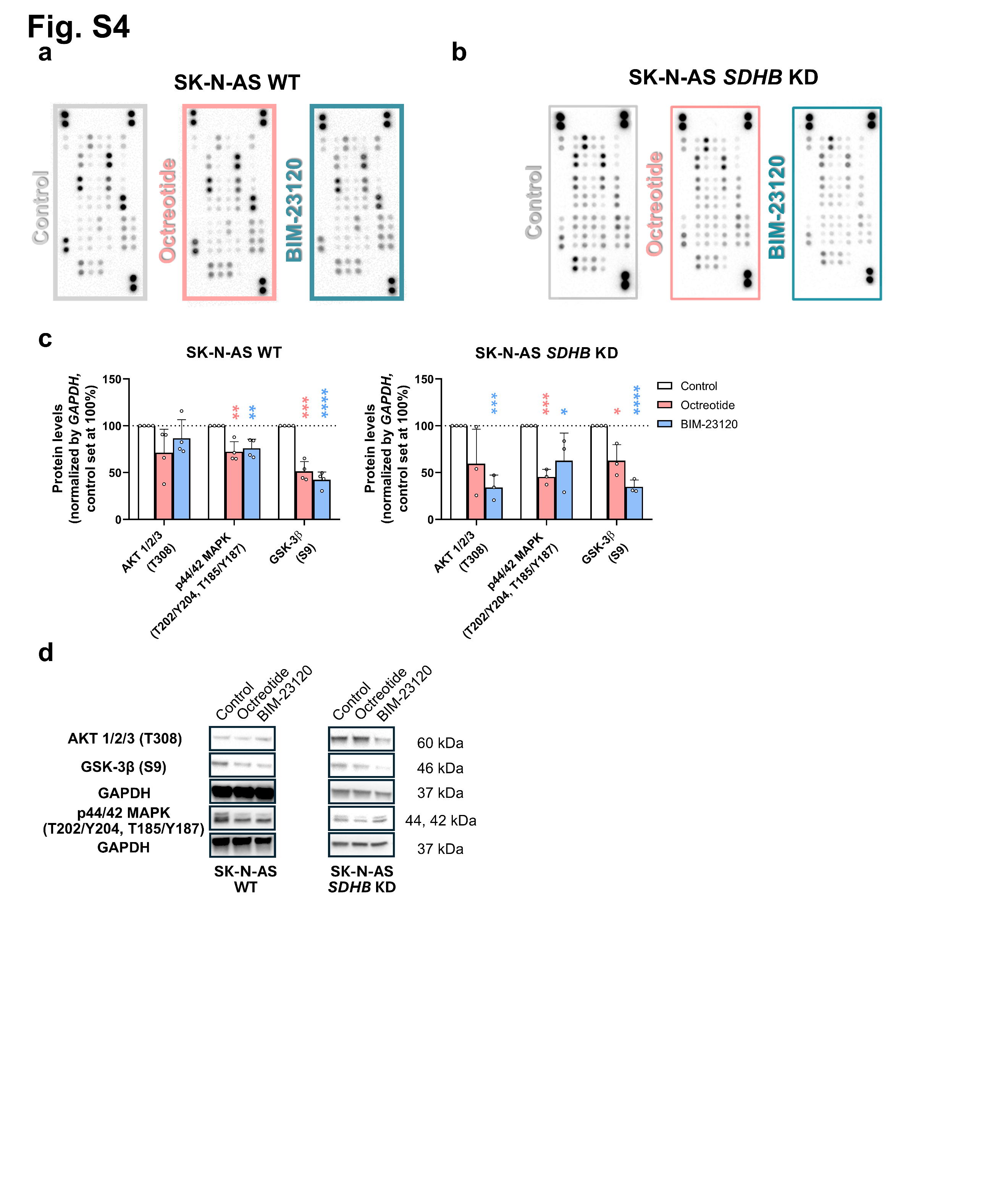


**Fig. S4** Validation of phosphoarray results by Western blot analysis. **a,b** Representative images of arrays treated with control (grey), octreotide (red) and BIM-23120 (blue) in the WT and *SDHB*-altered lines. **c,d** Validation by Western blot assay of p-AKT, p-p44/42 MAPK1/2 and p-GSK-3β phosphorylation in SK‑N‑AS WT and SK-N-AS *SDHB* KD (n = 4) with representative images, normalized by GAPDH. Colored asterisks indicate statistically significant differences compared with the control group (* *p* < 0.05, ** *p* < 0.01, *** *p* < 0.001, and **** *p* < 0.0001).

**
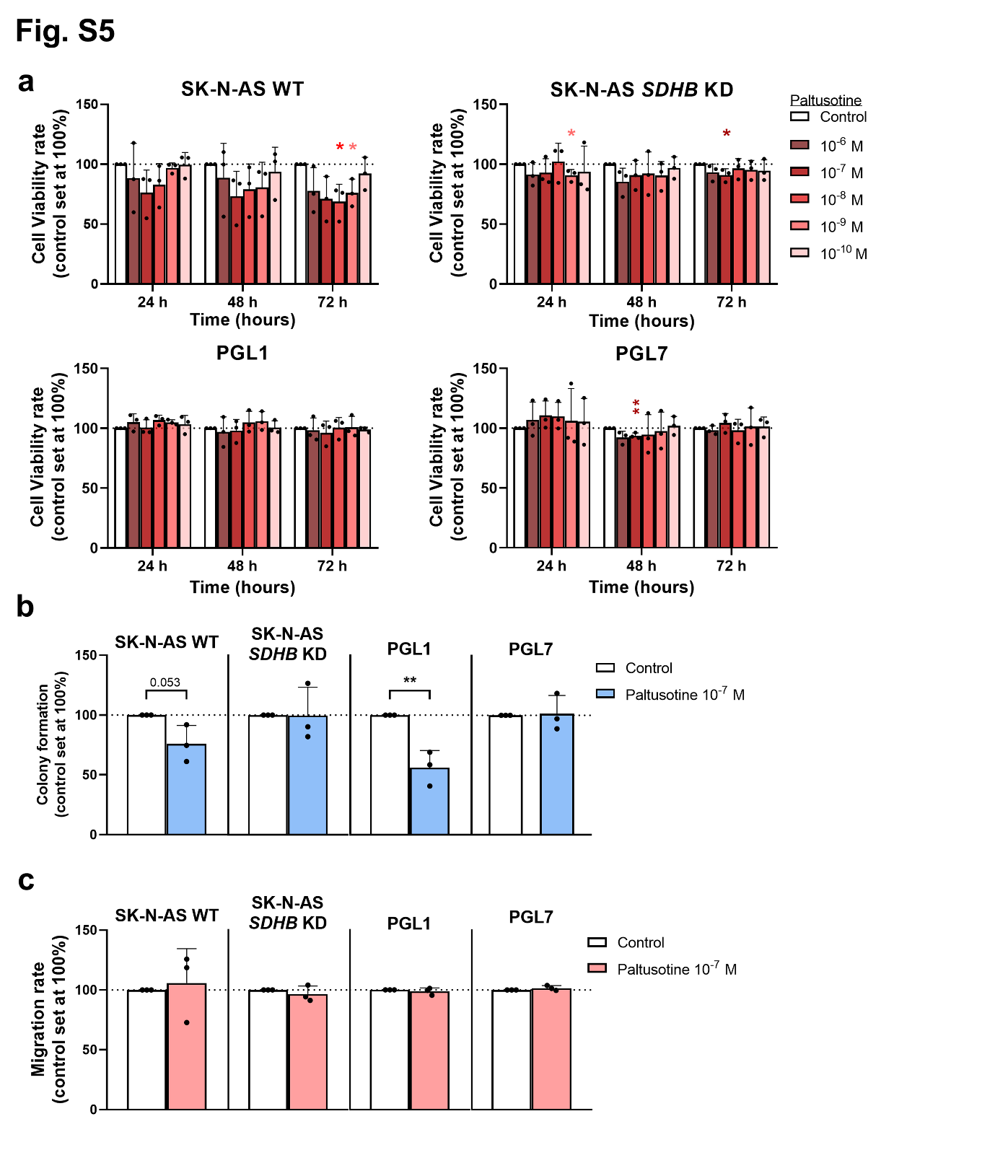
**

**Fig. S5** Paltusotine, a selective SST_2_ agonist, exerts moderate effects in PPGL cell models. **a** Effects on cell viability at 24, 48, and 72 h following paltusotine treatment at different concentrations in SK-N-AS WT (upper left), SK-N-AS *SDHB* KD (upper right), PGL1 (lower left), and PGL7 (lower right) cells (n = 3). **b** Changes in clonogenic capacity upon treatment with 10^-7^ M paltusotine in the aforementioned cell models (n = 3). **c** Effect of 10^-7^ M paltusotine on the migratory capacity of the same cell models (n = 3). Colored asterisks indicate statistically significant differences compared with the control group. Black asterisks above brackets denote statistically significant differences between the indicated groups (* *p* < 0.05, ** *p* < 0.01).

**References**

1. Calsina B, Piñeiro-Yáñez E, Martínez-Montes ÁM, Caleiras E, Fernández-Sanromán Á, Monteagudo M, et al. Genomic and immune landscape Of metastatic pheochromocytoma and paraganglioma. Nat Commun. 2023;14:1122. https://doi.org/10.1038/s41467-023-36769-6

2. Kim D, Pertea G, Trapnell C, Pimentel H, Kelley R, Salzberg SL. TopHat2: accurate alignment of transcriptomes in the presence of insertions, deletions and gene fusions. Genome Biol. 2013;14:R36. https://doi.org/10.1186/gb-2013-14-4-r36

3. Dobin A, Davis CA, Schlesinger F, Drenkow J, Zaleski C, Jha S, et al. STAR: ultrafast universal RNA-seq aligner. Bioinformatics. 2013;29:15–21. https://doi.org/10.1093/bioinformatics/bts635

4. Fishbein L, Leshchiner I, Walter V, Danilova L, Robertson AG, Johnson AR, et al. Comprehensive Molecular Characterization of Pheochromocytoma and Paraganglioma. Cancer Cell. 2017;31:181–93. https://doi.org/10.1016/j.ccell.2017.01.001

5. GTEx Consortium. The Genotype-Tissue Expression (GTEx) project. Nat Genet. 2013;45:580–5. https://doi.org/10.1038/ng.2653

6. Johnson WE, Li C, Rabinovic A. Adjusting batch effects in microarray expression data using empirical Bayes methods. Biostatistics. 2007;8:118–27. https://doi.org/10.1093/biostatistics/kxj037

7. Leek JT, Johnson WE, Parker HS, Jaffe AE, Storey JD. The sva package for removing batch effects and other unwanted variation in high-throughput experiments. Bioinformatics. 2012;28:882–3. https://doi.org/10.1093/bioinformatics/bts034

8. Rapizzi E, Fucci R, Giannoni E, Canu L, Richter S, Cirri P, et al. Role of microenvironment on neuroblastoma SK-N-AS SDHB-silenced cell metabolism and function. Endocr Relat Cancer. 2015;22:409–17. https://doi.org/10.1530/ERC-14-0479

9. Uphoff CC, Drexler HG. Detection of Mycoplasma contamination in cell cultures. Curr Protoc Mol Biol. 2014;106:28.4.1-28.4.14. https://doi.org/10.1002/0471142727.mb2804s106

10. Appetecchia M, Baldelli R. Somatostatin analogues in the treatment of gastroenteropancreatic neuroendocrine tumours, current aspects and new perspectives. J Exp Clin Cancer Res. 2010;29:19. https://doi.org/10.1186/1756-9966-29-19

11. Fukusumi S, Kitada C, Takekawa S, Kizawa H, Sakamoto J, Miyamoto M, et al. Identification and characterization of a novel human cortistatin-like peptide. Biochem Biophys Res Commun. 1997;232:157–63. https://doi.org/10.1006/bbrc.1997.6252

12. Gruszka A, Culler MD, Melmed S. Somatostatin analogs and chimeric somatostatin-dopamine molecules differentially regulate human growth hormone and prolactin gene expression and secretion in vitro. Mol Cell Endocrinol. 2012;362:104–9. https://doi.org/10.1016/j.mce.2012.05.020

13. Ren S-G, Taylor J, Dong J, Yu R, Culler MD, Melmed S. Functional association of somatostatin receptor subtypes 2 and 5 in inhibiting human growth hormone secretion. J Clin Endocrinol Metab. 2003;88:4239–45. https://doi.org/10.1210/jc.2003-030303

14. Ludvigsen E, Stridsberg M, Taylor JE, Culler MD, Öberg K, Janson ET. Subtype selective interactions of somatostatin and somatostatin analogs with sst1, sst2, and sst5 in BON-1 cells. Med Oncol. 2004;21:285–95. https://doi.org/10.1385/MO:21:3:285

15. Bruns C, Lewis I, Briner U, Meno-Tetang G, Weckbecker G. SOM230: a novel somatostatin peptidomimetic with broad somatotropin release inhibiting factor (SRIF) receptor binding and a unique antisecretory profile. Eur J Endocrinol. 2002;146:707–16. https://doi.org/10.1530/eje.0.1460707

16. Vázquez-Borrego MC, Gupta V, Ibáñez-Costa A, Gahete MD, Venegas-Moreno E, Toledano-Delgado Á, et al. A Somatostatin Receptor Subtype-3 (SST3) Peptide Agonist Shows Antitumor Effects in Experimental Models of Nonfunctioning Pituitary Tumors. Clin Cancer Res. 2020;26:957–69. https://doi.org/10.1158/1078-0432.CCR-19-2154

17. Zatelli MC, Piccin D, Tagliati F, Ambrosio MR, Margutti A, Padovani R, et al. Somatostatin receptor subtype 1 selective activation in human growth hormone (GH)- and prolactin (PRL)-secreting pituitary adenomas: effects on cell viability, GH, and PRL secretion. J Clin Endocrinol Metab. 2003;88:2797–802. https://doi.org/10.1210/jc.2002-021825

18. Gruszka A, Ren S-G, Dong J, Culler MD, Melmed S. Regulation of Growth Hormone and Prolactin Gene Expression and Secretion by Chimeric Somatostatin-Dopamine Molecules. Endocrinology. 2007;148:6107–14. https://doi.org/10.1210/en.2007-0378

19. Zatelli MC, Piccin D, Tagliati F, Bottoni A, Ambrosio MR, Margutti A, et al. Dopamine receptor subtype 2 and somatostatin receptor subtype 5 expression influences somatostatin analogs effects on human somatotroph pituitary adenomas in vitro. J Mol Endocrinol. 2005;35:333–41. https://doi.org/10.1677/jme.1.01876

20. Tagliati F, Zatelli MC, Bottoni A, Piccin D, Luchin A, Culler MD, et al. Role of Complex Cyclin D1/Cdk4 in Somatostatin Subtype 2 Receptor-Mediated Inhibition of Cell Proliferation of a Medullary Thyroid Carcinoma Cell Line in Vitro. Endocrinology. 2006;147:3530–8. https://doi.org/10.1210/en.2005-1479

21. Kiessling MK, Curioni-Fontecedro A, Samaras P, Lang S, Scharl M, Aguzzi A, et al. Targeting the mTOR Complex by Everolimus in NRAS Mutant Neuroblastoma. PLoS One. 2016;11:e0147682. https://doi.org/10.1371/journal.pone.0147682

22. Calero R, Morchon E, Johnsen JI, Serrano R. Sunitinib Suppress Neuroblastoma Growth through Degradation of MYCN and Inhibition of Angiogenesis. PLoS One. 2014;9:e95628. https://doi.org/10.1371/journal.pone.0095628

23. Zhao J, Wang S, Markison S, Kim SH, Han S, Chen M, et al. Discovery of Paltusotine (CRN00808), a Potent, Selective, and Orally Bioavailable Non-peptide SST2 Agonist. ACS Med Chem Lett. 2022;14:66–74. https://doi.org/10.1021/acsmedchemlett.2c00431

24. Moreno-Montilla MT, Pedraza-Arevalo S, Martínez-López A, Blázquez-Encinas R, García-Vioque V, Rodríguez-Ortiz L, et al. Exploring RNA biology in pseudomyxoma peritonei uncovers splicing dysregulation as a novel, targetable molecular vulnerability. Cancer Gene Ther. 2025;32:721–36. https://doi.org/10.1038/s41417-025-00911-x

25. Vandesompele J, De Preter K, Pattyn F, Poppe B, Van Roy N, De Paepe A, et al. Accurate normalization of real-time quantitative RT-PCR data by geometric averaging of multiple internal control genes. Genome Biol. 2002;3:RESEARCH0034. https://doi.org/10.1186/gb-2002-3-7-research0034

26. Alors-Pérez E, Pedraza-Arevalo S, Blázquez-Encinas R, García-Vioque V, Agraz-Doblas A, Yubero-Serrano EM, et al. Altered CELF4 splicing factor enhances pancreatic neuroendocrine tumors aggressiveness influencing mTOR and everolimus response. Mol Ther Nucleic Acids. 2024;35:102090. https://doi.org/10.1016/j.omtn.2023.102090

27. Pang Z, Lu Y, Zhou G, Hui F, Xu L, Viau C, et al. MetaboAnalyst 6.0: towards a unified platform for metabolomics data processing, analysis and interpretation. Nucleic Acids Res. 2024;52:W398–406. https://doi.org/10.1093/nar/gkae253
